# Redox-Switchable Chalcogen Bonding for Anion Recognition and Sensing

**DOI:** 10.1021/jacs.2c02924

**Published:** 2022-05-06

**Authors:** Robert Hein, Andrew Docker, Jason J. Davis, Paul D. Beer

**Affiliations:** † Department of Chemistry, Chemistry Research Laboratory, University of Oxford, Mansfield Road, Oxford OX1 3TA, U.K.; ‡ Department of Chemistry, Physical & Theoretical Chemistry Laboratory, South Parks Road, Oxford OX1 3QZ, U.K.

## Abstract

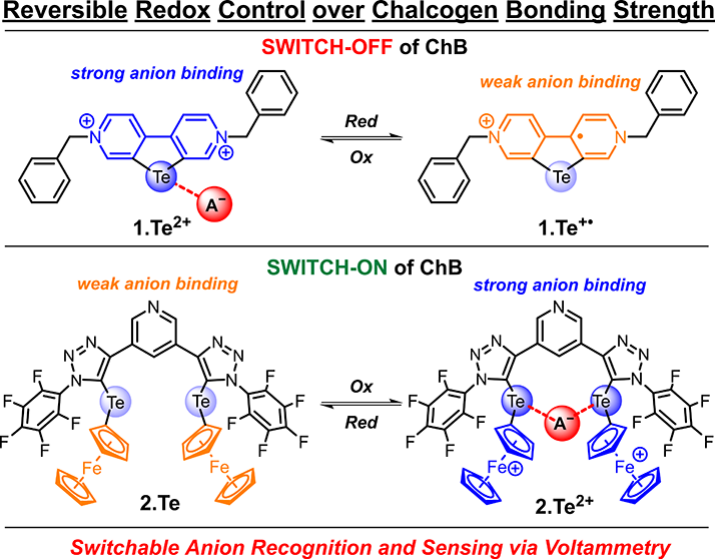

Inspired by the success of its related sigma-hole congener halogen bonding (XB), chalcogen bonding (ChB) is emerging as a powerful noncovalent interaction with a plethora of applications in supramolecular chemistry and beyond. Despite its increasing importance, the judicious modulation of ChB donor strength remains a formidable challenge. Herein, we present, for the first time, the reversible and large-scale modulation of ChB potency by electrochemical redox control. This is exemplified by both the switching-ON of anion recognition via ChB oxidative activation of a novel bis(ferrocenyltellurotriazole) anion host and switching-OFF reductive ChB deactivation of anion binding potency with a telluroviologen receptor. The direct linking of the redox-active center and ChB receptor donor sites enables strong coupling, which is reflected by up to a remarkable 3 orders of magnitude modulation of anion binding strength. This is demonstrated through large voltammetric perturbations of the respective receptor ferrocene and viologen redox couples, enabling, for the first time, ChB-mediated electrochemical anion sensing. The sensors not only display significant anion-binding-induced electrochemical responses in competitive aqueous-organic solvent systems but can compete with, or even outperform similar, highly potent XB and HB sensors. These observations serve to highlight a unique (redox) tunability of ChB and pave the way for further exploration of the reversible (redox) modulation of ChB in a wide range of applications, including anion sensors as well as molecular switches and machines.

## Introduction

Sigma-hole interactions, in particular halogen bonding (XB) and more recently chalcogen bonding (ChB), defined as the attractive interaction between Lewis bases and an electron-deficient region of a group 17 and group 16 atom, respectively, have emerged as highly potent and versatile noncovalent interactions.^1^ Their utility is becoming well-established across diverse fields, including crystal engineering,^2,3^ materials chemistry,^4−9^ organocatalysis,^10−15^ and anion supramolecular chemistry.^16−20^ The latter includes anion recognition,^21−24^ transport,^25−29^ and sensing,^28,30−39^ wherein a notably enhanced performance of sigma-hole-based anion receptors, in particular XB hosts, in comparison to their traditional hydrogen bonding (HB) analogues is well-documented. This includes enhanced anion binding affinities, selectivities, transport efficacies, and sensory performances, attributable to the more stringent linearity requirements of the electron-deficient XB/ChB donor atom—anion interaction as well as their unique electronic properties and tunability.^19,40−42^

Indeed, recent reports have demonstrated that the ChB donor potency is highly sensitive to the local electronic environment and can be modulated through either covalent substituent variation^24,26,42,43^ or heteroditopic ion-pair recognition.^44^ In the context of anion recognition, this tunable ChB donor potency provides an attractive strategy to generate stimuli-responsive host systems. For example, Gabbaï and co-workers have recently reported the notable increased potency of Te(IV) cations as ChB anion receptors and transporters in comparison to their parent, neutral Te(II) analogues.^26^ This was achieved by the oxidative methylation of diaryltellurides, a powerful, but irreversible, means of tuning the ChB donor properties.

Conceivably, control of local ChB donor potency could also be achieved by coupling of a chalcogen center to a redox-active moiety, wherein oxidation or reduction reversibly switches-ON or -OFF chalcogen centered electrophilicity.

While thus far unprecedented for ChB, the reversible (electrochemical) redox control over XB donor strength is already established, in particular with XB ferrocene and TTF receptor-based systems.^32,33,45−49^ This is saliently reflected in an associated cathodic perturbation of the redox potential of the electroactive XB receptor in the presence of Lewis bases, thereby presenting a simple, yet powerful, means of electrochemical anion detection, as increasingly exploited in sensors.^32,36,37,39^

**Figure 1:**
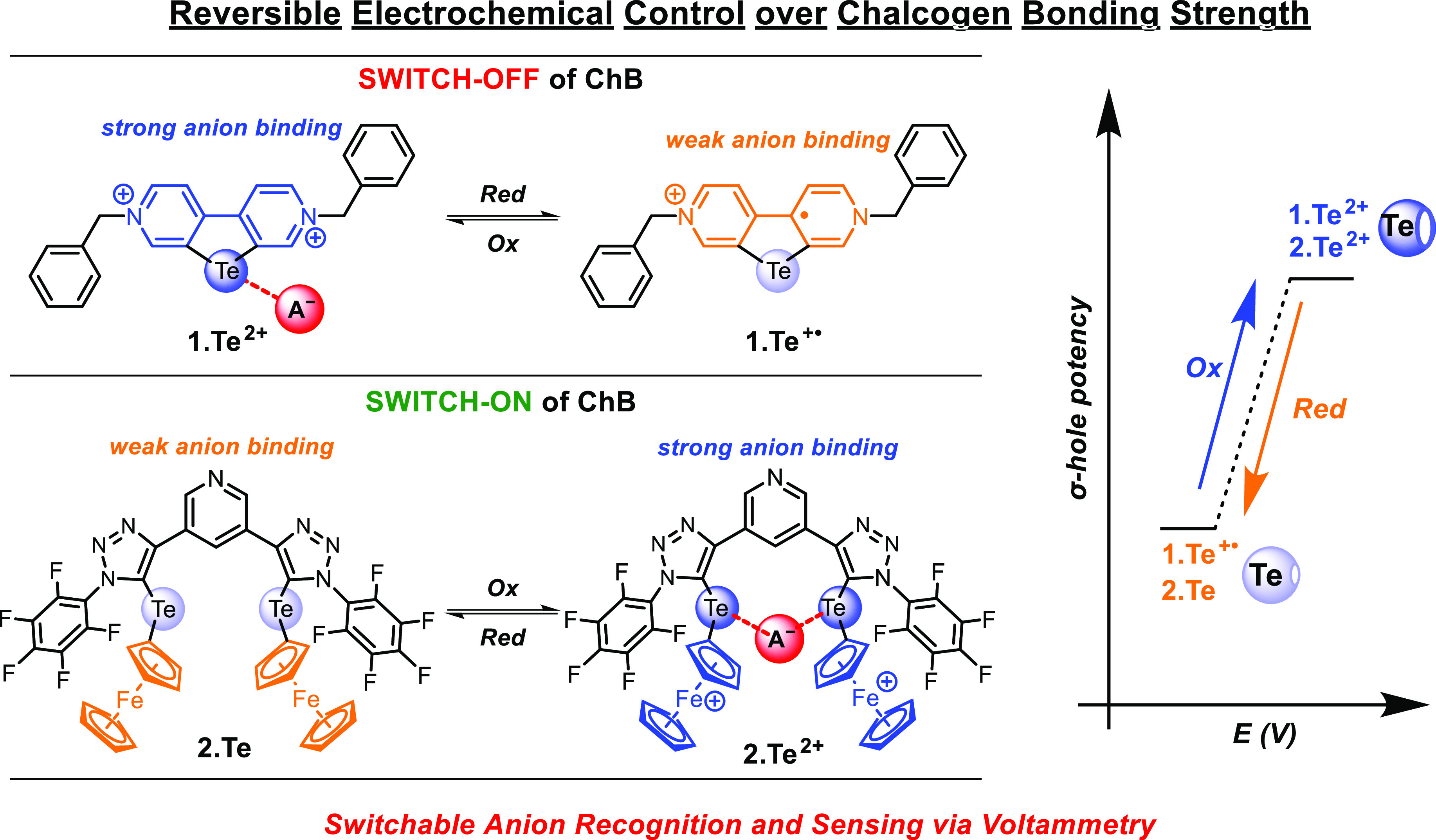
Illustrative overview of the redox control of chalcogen bonding (ChB) potency. We demonstrate both reversible electrochemical reductive deactivation of ChB in telluroviologens as well as oxidative activation of ChB in ferrocenyltellurotriazole receptors.

Given the compelling evidence for the tunable nature of ChB interactions, it was envisaged that the construction of redox-responsive ChB receptor systems would facilitate a potentially powerful means to electrochemically modulate sigma-hole donor potency with high degrees of ON–OFF state fidelity. To explore this concept, we investigated two redox-active chalcogen-containing anion receptor motifs, namely, chalcogenoviologens and a bis(ferrocenyltellurotriazole), wherein the ChB donor potency can be reversibly switched-OFF or -ON by redox control (Figure 1).

In the case of the natively dicationic chalcogenoviologens, we demonstrate a complete deactivation of ChB sigma-hole donor strength and associated anion binding affinity upon electrochemical reduction. Conversely, an activation of anion binding potency of up to 3-orders of magnitude is achieved by oxidation of the neutral ChB bis(ferrocenyltellurotriazole) host system. Not only does this provide a powerful and unprecedented means of reversible control over ChB donor strength, but also enables highly sensitive voltammetric anion detection. This work thereby constitutes a very rare example of ChB-mediated sensing^28,38^ and the first exploitation of ChB in electrochemical sensing.

## Results and Discussion

### Synthesis of Redox-Active ChB Anion Receptors

**Scheme 1:**
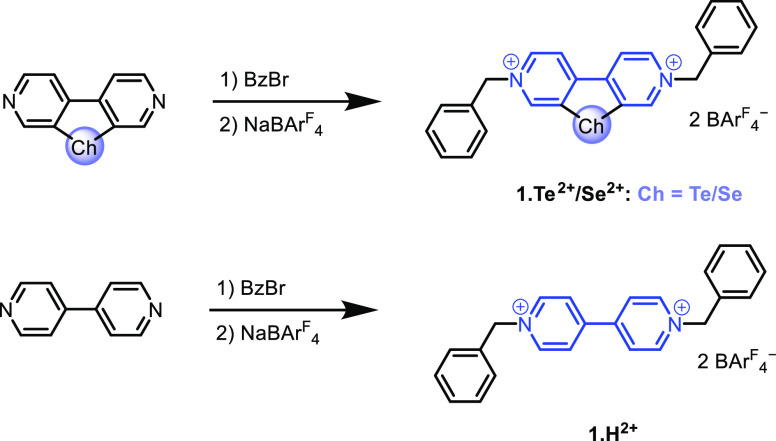
Synthetic Route for Dicationic Redox-Active Chalcogenoviologen Hosts **1.Te^2+^** and **1.Se^2+^** as well as the Control Proto-Congener **1.H^2+^**

The synthesis of the chalcogenoviologens **1.Te^2+^** and **1.Se^2+^** was achieved via reaction of the chalcogen-functionalized bipyridine precursors^50^ with benzyl bromide, followed by salt metathesis with sodium tetrakis(3,5-bis(trifluoromethyl)phenyl)borate (NaBAr^F^_4_), to afford the target receptors **1.Te^2+^** and **1.Se^2+^**, in yields of 61% and 57%, respectively (Scheme 1). In addition, for the purposes of delineating the role of nonspecific electrostatic interactions in the anion sensing behavior of the viologen systems, an unfunctionalized derivative **1.H^2+^** was also prepared.

**Scheme 2:**
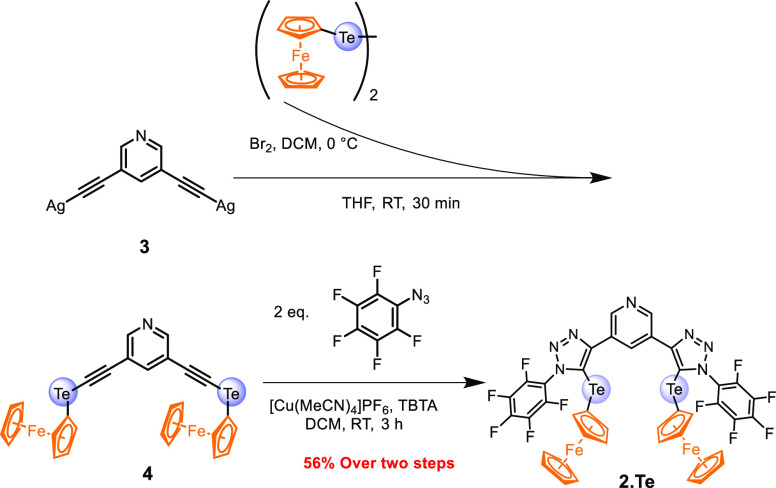
Synthetic Route for Neutral Redox-Active Pyridine Bis-(ferrocenyltellurotriazole) Receptor **2.Te**

The novel ChB bis(ferrocene-telluro-triazole) receptor **2.Te** was constructed by direct appendage of ferrocene (Fc) redox reporters onto an established bis(tellurotriazole) anion receptive scaffold (Scheme 2).^25,42,44^ This was achieved by reaction of pyridine bis(silveracetylide)^42^**3** with in situ generated ferrocenyl tellurobromide, obtained from reaction of diferrocenyl ditelluride^51^ with bromine, affording 3,5-pyridine bis(ferrocenylalkyne) **4**. This compound was immediately subjected to copper(I)-catalyzed azide-alkyne cycloaddition (CuAAC)^22^ with two equivalents of azido pentafluorobenzene^52^ affording the title compound **2.Te** in 54% yield over two steps.^53^ Further detail and full compound characterization of all receptors by ^1^H and ^13^C NMR as well as ESI-MS, can be found in the Supporting Information.

### ^1^H NMR and UV–Vis Anion Binding Studies

**Figure 2:**
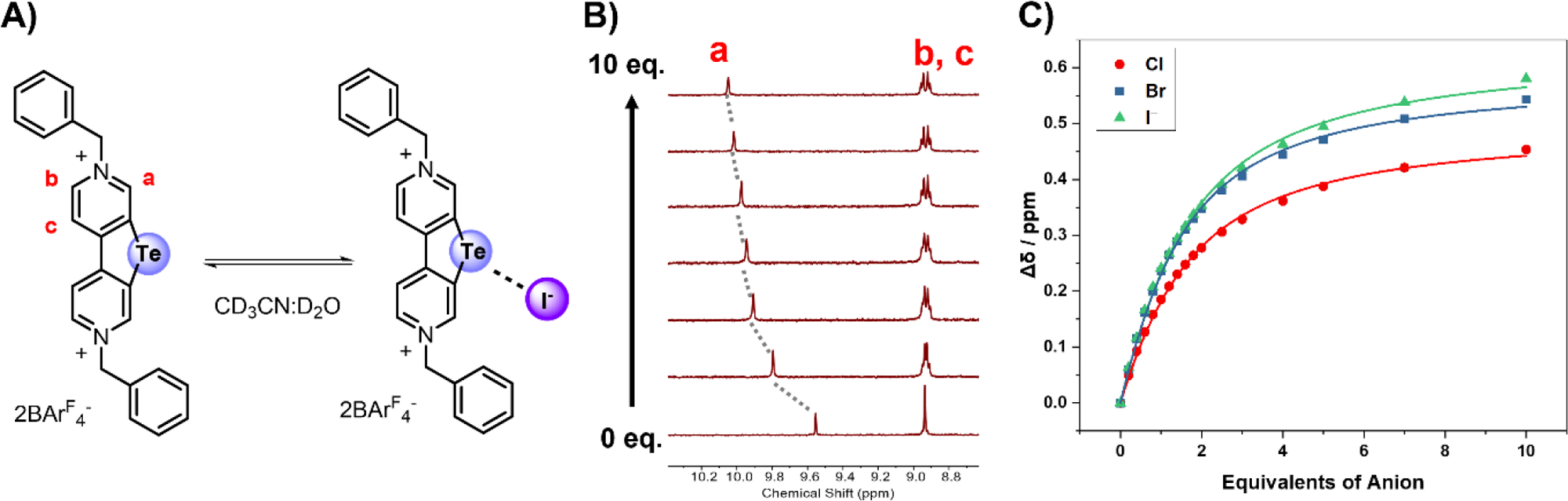
(A) Proposed anion binding mode of **1.Te^2+^** with iodide as a representative halide anion. (B) Representative ^1^H NMR chemical shift perturbations of protons *a–c* of **1.Te^2+^** upon titration with TBA-iodide in CD_3_CN/D_2_O 9:1. (C) Corresponding halide anion binding isotherms.

The ChB donor halide anion binding capabilities of the natively dicationic viologen receptors **1.Te^2+^** and **1.Se^2+^** in their switch-ON state were initially investigated via ^1^H NMR titrations in CD_3_CN/D_2_O 9:1. This competitive aqueous solvent system was chosen to ensure the solubility of the charged ChB hosts and their anion complexes. As representatively shown for the titration of **1.Te^2+^** with iodide, the addition of all halide anions as their tetrabutylammonium (TBA) salts induced notable chemical shift perturbations of the proximal viologen proton *a* for both **1.Te^2+^/Se^2+^**, in excellent agreement with ChB participation as schematically shown in Figure 2A,B.

Analysis of the corresponding binding isotherms (Figure 2C) revealed moderately strong 1:1 host–guest stoichiometric binding with association constants *K*_a_ of up to 1036 M^–1^ for Br^–^ recognition with **1.Te^2+^** (Table 1).^54^ Both Cl^–^ and I^–^ displayed similarly strong binding to **1.Te^2+^** of ≈880 M^–1^. Notably, the affinity of all halides is, as expected, markedly lower for the lighter, less polarizable ChB analogue **1.Se^2+^** with *K*_a_ ≈ 180 M^–1^. Furthermore, the proto viologen analogue **1.H^2+^** displays even weaker halide affinities of up to *K*_a_ = 139 M^–1^ for Br^–^.

**Table 1:** Anion Association Constants *K* (M^–1^) of 1.Te^2+^/Se^2+^/H^2+^ as Determined by ^1^H NMR Titrations in CD_3_CN/D_2_O 9:1

	**1.Te^2+^**	**1.Se^2+^**	**1.H^2+^**
Cl^–^	877 ± 3	181 ± 1	127 ± 1
Br^–^	1036 ± 3	182 ± 2	139 ± 1
I^–^	888 ± 4	179 ± 3	130 ± 1

These observations importantly confirm ChB participation in anion binding, especially for **1.Te^2+^** and to a lesser extent **1.Se^2+^**, and highlight that halide anion recognition by **1.Te^2+^/Se^2+^** is not driven by electrostatic or HB interactions alone. Of further note is the modest bromide selectivity observed for **1.Te^2+^**, while neither **1.Se^2+^** nor **1.H^2+^** exhibits a halide preference.

Having established the ChB potency of the dicationic **1.Te^2+^**, attention turned to the assessment of the anion binding capability of the natively neutral bis(ferrocenyltellurotriazole) **2.Te** receptor in the switch-OFF state.^55^ Analogous ^1^H NMR anion binding studies revealed, as expected with the neutral receptor, that the ChB potency was strongly diminished.

Indeed, no measurable ^1^H NMR perturbations for a range of halide or oxoanion guests were observed even in the much less competitive d_6_-acetone. Only the addition of H_2_PO_4_^–^ induced significant chemical shift perturbations of the internal pyridine proton of **2.Te**, wherein analysis of the binding isotherm determined a weak 1:1 host–guest stoichiometric association constant of 111 M^–1^ (Figure S12 and Table S1). Structurally related pyridine bis(tellurotriazole) ChB receptors typically display stronger anion binding in this solvent,^42^ indicating a somewhat decreased neutral ChB potency of **2.Te**, presumably arising due to a combination of an increased electron density at the chalcogen center and steric constraints from the large and comparably electron-rich ferrocene substituents.

These findings support a very weak ChB sigma-hole donor capability of the neutral **2.Te** receptor (ChB OFF) in acetone and acetonitrile, while the dicationic **1.Te^2+^/Se^2+^** receptor systems expectedly display much improved native anion binding capabilities even in competitive aqueous media (CD_3_CN/D_2_O 9:1; ChB ON), providing a first indication of the strong dependence of ChB sigma-hole donor strength on the respective receptor’s redox state.

**Figure 3:**
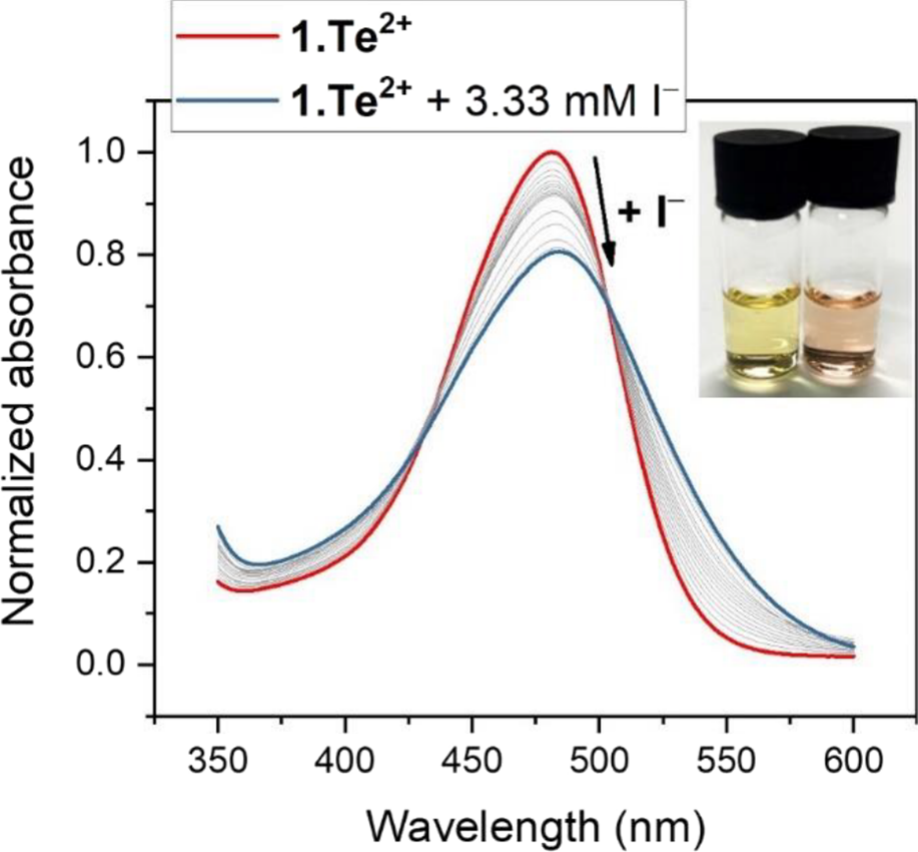
Normalized UV–vis spectra of **1.Te^2+^** (100 μM) upon titration with up to 3.33 mM iodide in CH_3_CN/H_2_O 9:1. The inset shows the color of the receptor in the absence (left, yellow) and presence of 3.33 mM I^–^ (right, red).

The significant ChB anion recognition potency of **1.Te^2+^** was further supported by UV–vis anion binding studies in the same solvent system as employed for ^1^H NMR studies. In CH_3_CN/H_2_O 9:1, **1.Te^2+^** displays significant absorbance changes upon titration with halide anions, whereby, in the presence of iodide, the absorbance band at λ_max_ = 481 nm shifts bathochromically with appearance of an isosbestic point at 505 nm (Figures 3 and S13–S15). These perturbations are large enough to be detectable by the naked eye (change from yellow to red) and present a rare example of ChB-mediated optical anion sensing.^7,24,28,56,57^

Pleasingly, the 1:1 host–guest stoichiometric binding constants obtained by global fitting of the UV–vis titration data are in good agreement with those obtained by ^1^H NMR titrations; Br^–^ is bound strongest with *K* = 873 M^–1^, while binding of Cl^–^ and I^–^ is slightly weaker with 690 and 627 M^–1^, respectively (Figures S13–S15).

Furthermore, **1.Se^2+^** displays only minor UV–vis perturbations toward halide anions (Figure S16), too small to accurately quantify. Similarly, and again in good agreement with ^1^H NMR studies, the proto analogue **1.H^2+^** is neither colored nor optically responsive to anions (Figure S17).

### Electrochemical Characterization of Receptors

**Figure 4:**
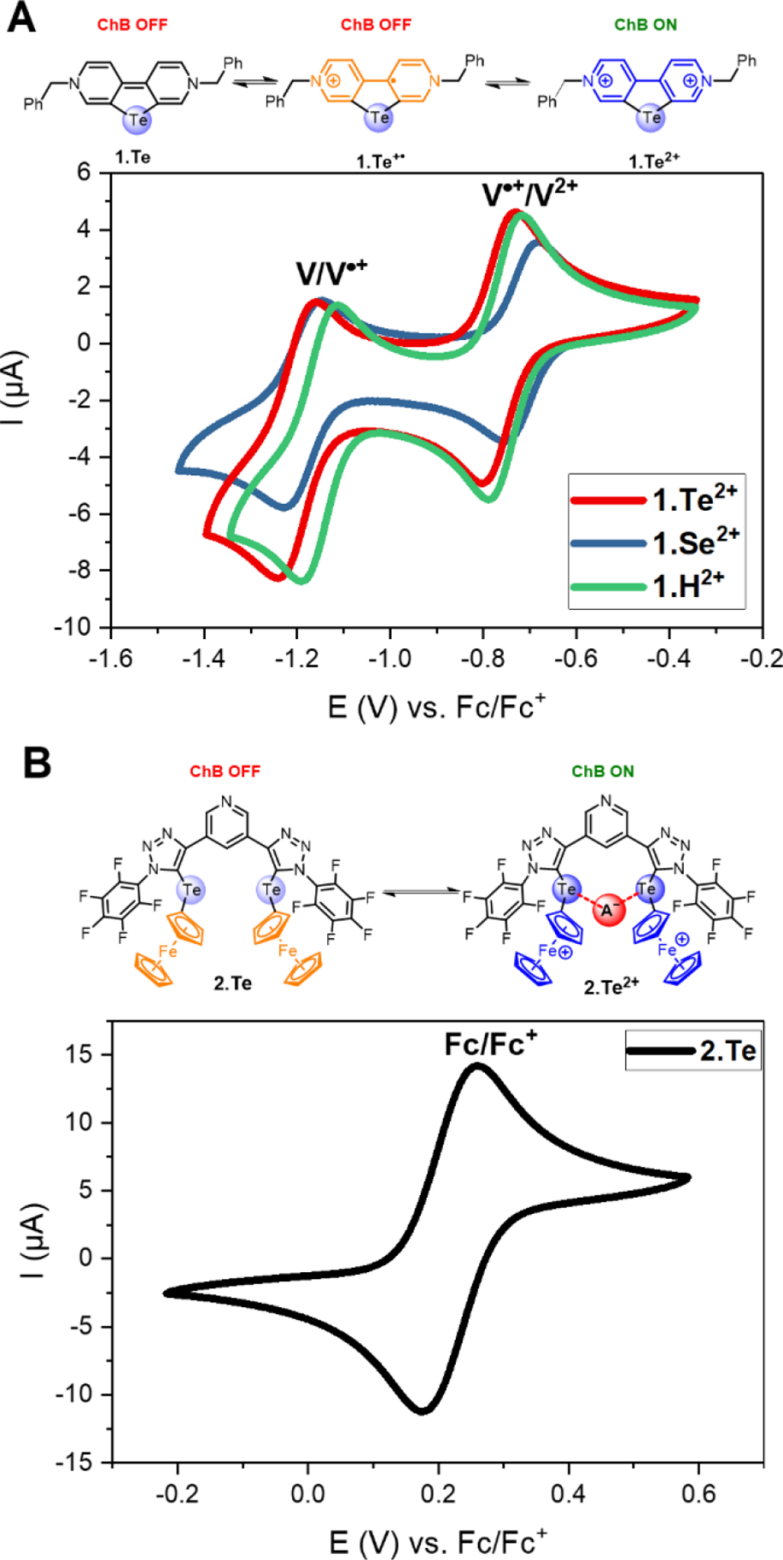
(A) CVs of **1.Te^2+^/Se^2+^/H^2+^** in degassed CH_3_CN /H_2_O 9:1. (B) CVs of **2.Te** in CH_3_CN. All CVs were recorded at a scan rate of 100 mV/s in the presence of 0.25 mM receptor and 100 mM TBAPF_6_ supporting electrolyte. Shown are also the different receptor oxidation states.

The electrochemical characterization of all the ChB redox-active receptors was carried out by cyclic voltammetry (CV) and square-wave voltammetry (SWV) in the presence of 100 mM TBAPF_6_ as the supporting electrolyte. For solubility reasons, **1.Te^2+^/Se^2+^/H^2+^** were only studied in the same solvent system of CH_3_CN/H_2_O 9:1 as employed for ^1^H NMR and UV–vis studies. As shown in Figure 4A, all the viologen receptors displayed two well-defined, one-electron reductive couples (V^2+^/V^•+^ and V^•+^/V) at similar, moderately cathodic potentials (Table S2), in good agreement with related previous studies.^50,58^ Both couples display a high degree of reversibility as evidenced by a close-to-unity ratio of anodic and cathodic peak currents (*i*_pa_/*i*_pc_) and excellent linearity of these currents with the square root of the scan rate (Figures S18–S20), confirming a diffusion-controlled process. These observations highlight that introduction of the chalcogen donor atom into the viologen scaffold does not significantly affect its electrochemical properties. For example, the half-wave potentials *E*_1/2_ of all three viologens are very similar and lie between −0.720 and −0.770 V for the first reductive couple (V^2+^/V^•+^) and between −1.160 and −1.210 V for the second reductive couple (V^•+^/V) (Table S2).

The improved solubility of neutral **2.Te** in comparison to dicationic **1.Te^2+^** enabled electrochemical studies across a wider range of solvent systems. In all tested solvents, DCM, acetone, CH_3_CN (Figure 4B), and MeOH containing 100 mM TBAPF_6_ supporting electrolyte, **2.Te** displayed a single, well-defined, reversible, diffusion-controlled redox couple (see also Figures S21–24). This suggests that both Fc motifs undergo one-electron oxidation simultaneously and independently of one another, generating the dicationic **2.Te^2+^**. Only in the presence of the much larger, less-coordinating BAr^F^_4_^–^ anion as the electrolyte in DCM, was electronic communication between the Fc motifs observed, as evidenced by appearance of two separate redox couples with a peak separation of ≈90 mV (Figures S25 and S26).^59^

As a result of the strongly electron-withdrawing perfluorobenzene appendages, the receptor’s half-wave potential *E*_1/2_ = 0.218 V in CH_3_CN, is significantly more positive than that of parent Fc (0 V) (Table S3).

### Reductive Switch-OFF Deactivation of ChB in Telluroviologen

**Figure 5:**
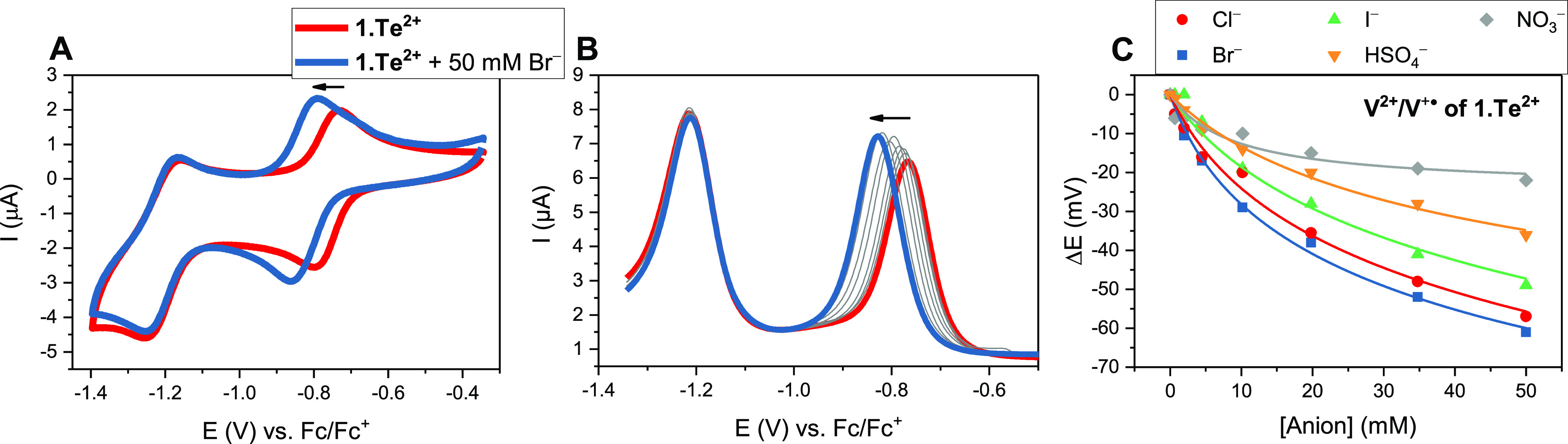
(A) CVs and (B) SWVs of 0.1 mM **1.Te^2+^** in degassed CH_3_CN/H_2_O 9:1 and 100 mM TBAPF_6_ upon titration with up to 50 mM bromide. (C) Voltammetric binding responses of the first reductive couple (V^•+^/V^2+^) of 0.1 mM **1.Te^2+^** in the same solvent upon titration with various anions. Solid lines represent fits according to the 1:1 host–guest Nernst model (eq 1). All titrations were carried out at constant host concentration and ionic strength.

In order to assess the ChB donor strength of the redox-active receptors, we conducted voltammetric anion sensing studies. This provides a particularly convenient and straight-forward means of assessing the ChB potency, as the magnitude of voltammetric perturbation in the presence of the analyte is directly dependent on its binding strength to the different receptor oxidation states, as discussed in more detail below. As shown in Figure 5, the first reductive couple (V^•+^/V^2+^) of **1.Te^2+^** displayed significant, continuous cathodic voltammetric shifts toward all tested anions in CH_3_CN/H_2_O 9:1, with a notable preference for the halides according to the following response selectivity, defined by the maximum cathodic voltammetric perturbation Δ*E*_max_: Br^–^ > Cl^–^ > I^–^ > HSO_4_^–^ > NO_3_^–^. Bromide induced the largest cathodic shifts of up to −61 mV in the presence of 50 mM anion (Table 2), with an almost identical shift observed for Cl^–^ of up to −57 mV, while the response toward I^–^ was smaller (−49 mV). Both oxoanions hydrogen sulfate and nitrate displayed an attenuated response of −36 and −22 mV, respectively. The initial equivalent additions of H_2_PO_4_^–^ also induced significant cathodic perturbations (≥50 mV); however, precipitation problems precluded quantitative binding data being determined. In contrast, the second reductive couple did not display significant perturbations in the presence of any anion.

**Table 2:** Maximum Cathodic Voltammetric Shifts Δ*E*_max_ of **1.Te^2+^**/**Se^2+^**/**H^2+^**, in the Presence of 50 mM Anion, and *K*_Ox_ = *K*_1.Te2+/Se2+_ as Determined by Fitting of the Voltammetric Binding Isotherms According to Eq 1, for the First Reductive Couple of Viologen Hosts toward Various Anions in Degassed CH_3_CN/H_2_O 9:1 and 100 mM TBAPF_6_

	**1.Te^2+^**			**1.Se^2+^**		**1.H^2+^**	
anion	Δ*E*_max_ (mV)^a^	*K*_1.Te2+_ (M^–1^)^b^	BEF^c^	Δ*E*_max_ (mV)^a^	*K*_1.Se2+_ (M^–1^)^b^	Δ*E*_max_ (mV)^a^	*K*_1.H2+_ (M^–1^)^b^
Cl^–^	–57	156	9.3	–22	26	–17	n/a
Br^–^	–61	205	10.8	–15	n/a	–13	n/a
I^–^	–49	107	6.8	–16	n/a	/	
HSO_4_^–^	–36	77	4.1	/		/	
NO_3_^–^	–22	n/a	n/a	/		/	

a Estimated error ± 3 mV.

b *K*_Red_ = *K*_1.Te+•/Se+•_ is ≈ 0 in all cases.

c Binding enhancement factor (BEF = *K*_1.Te2+/Se2+_/*K*_1.Te•+/Se•+_), calculated from Δ*E*_max_ according to eq S2, see the Supporting Information for further details. /– not conducted. n/a – not meaningful due to small response.

Selected comparative halide sensing studies were also carried out with **1.Se^2+^** and **1.H^2+^**. Both responded weakly, with the largest cathodic perturbation observed for **1.Se^2+^** in the presence of Cl^–^ (−22 mV), while the response of **1.H^2+^** toward Cl^–^ and Br^–^ was even smaller (−17 and −13 mV, respectively, see Table 2 and Figure S27). These observations are in excellent agreement with the ^1^H NMR and UV–vis binding studies and crucially confirm that the anion binding and sensing performance of **1.Te^2+^** is mediated by ChB interactions via the Te-donor atoms.

The significant cathodic response magnitudes observed for **1.Te^2+^** arise as a result of anion recognition-induced stabilization of the native, oxidized redox state. The response magnitude Δ*E* is dependent on the ratio of the anion binding constants to the native, oxidized **1.Te^2+^**/**Se^2+^** (*K*_Ox_ = *K*_**1.Te2+/Se2+**_) and the monoreduced **1.Te**^•+^/**Se**^•+^ (*K*_Red_ = *K***_1.Te•+/Se•+_)** receptor states, and is, according to eq 1, directly proportional to their ratio (*K*_Ox_/*K*_Red_), the binding-enhancement factor (BEF).^39^

The absolute values of both *K*_Ox_ and *K*_Red_ were obtained by fitting of the voltammetric response isotherms (Figure 5C) according to the established 1:1 host–guest stoichiometric Nernst model (eq 1).^33,39,46,49^
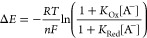
1

As shown in Table 2, *K*_**1.Te2+**_ with the halides and bisulfate is moderate but significant (77–205 M^–1^) and the largest for Br^–^. Pleasingly, this is in good agreement with ^1^H NMR binding studies where the strongest Br^–^ binding was observed (Table 1). The somewhat smaller magnitude of *K*_Ox_ determined voltammetrically in comparison to the analogous ^1^H NMR binding constants is in line with similar prior reports and most likely arises from electrolyte effects as detailed elsewhere.^60^

In all cases, *K*_**1.Te•+/Se•+**_, that is binding to the monocationic **1.Te**^•+^/**Se**^•+^, is negligible (*K*_**1.Te•+/Se•+**_ ≈ 0 M^–1^), indicative of complete ChB switch-OFF and anion decomplexation from the **1.Te^2+^** host upon monoreduction to **1.Te^•+^**. This is in excellent agreement with negligible voltammetric perturbations observed for the second reductive couple; i.e., in this solvent system, monoreduction is sufficient to completely deactivate ChB-mediated anion binding, such that a further reduction to **1.Te** has no additional impact on anion binding strength.^61^

We further quantified the redox switching magnitude of **1.Te^2+^** by calculating the BEF = *K*_**1.Te2+**_/*K*_**1.Te•+**_ according to a simplified model (BEF =  (eq S2), see Table 2, refs,^33,39^ and SI Section 6 for further details). This confirmed significant ChB binding modulation (i.e., deactivation) by a factor of up to ≈11-fold in the case of bromide.

Importantly, the redox reversibility of both redox couples of the telluroviologen host is generally maintained in the presence of anions, highlighting that ChB potency and anion binding can be judiciously reversibly switched OFF by redox control. As discussed in more detail in a later section, the sensory performance of this simple ChB voltammetric anion sensing system and redox-switch compares very favorably with those of a range of more elaborate XB or HB redox active systems (see Figure S25).

### Oxidative Switch-ON Activation of ChB in Ferrocenyltellurotriazole **2.Te**

Having established the general utility of (reductive) redox-controlled ChB in simple telluroviologens, our attention focused on a further elaboration of this concept in the more advanced anion receptor scaffold **2.Te**. The ChB potency of this natively neutral receptor can be reversibly switched-ON by redox control of the ferrocene/ferrocenium (Fc/Fc^+^) couple, a more common, and arguably more useful approach for anion recognition and sensing, circumventing the isolation of charged compounds.^36,39,47,62,63^

**Figure 6:**
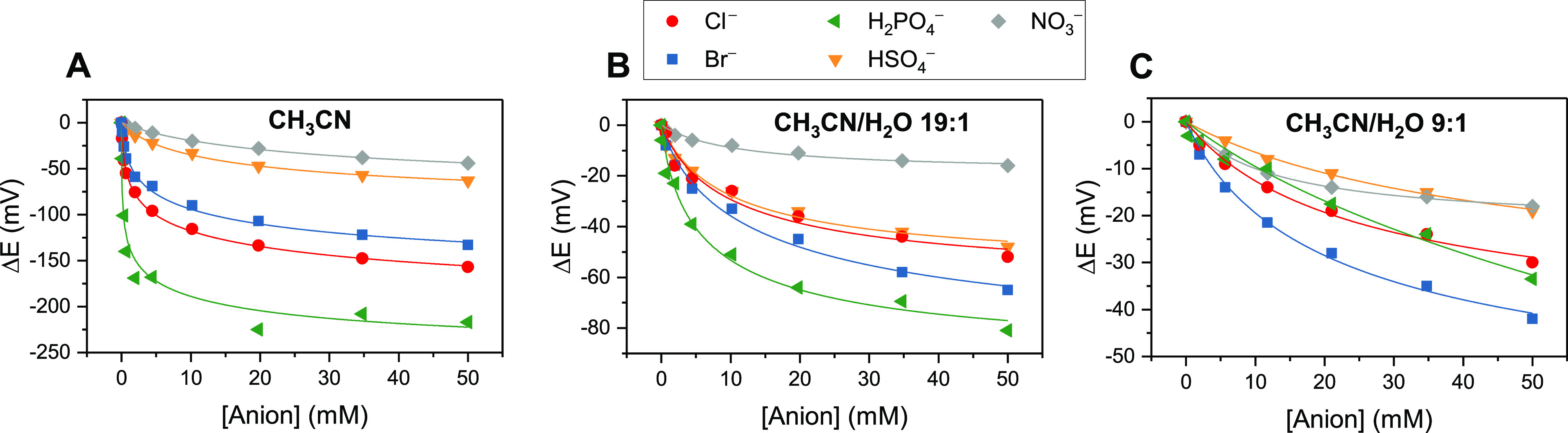
Voltammetric binding responses of **2.Te** in (A) CH_3_CN, (B) CH_3_CN/H_2_O 19:1 and (C) CH_3_CN/H_2_O 9:1 upon titration with various anions. [**2.Te**] = 0.1 mM with 100 mM TBAPF_6_ supporting electrolyte. The overall ionic strength was kept constant at 100 mM throughout. Solid lines represent fits to a 1:1 host–guest Nernst model eq 1. Note the different *y* axis scaling for the graphs.

To assess this capability, voltammetric sensing studies with halides and oxoanions were undertaken. Due to the improved solubility of neutral **2.Te**, these were conducted in a range of solvent systems. In CH_3_CN, **2.Te** voltammetrically responded to all tested anions (H_2_PO_4_^–^, HSO_4_^–^, NO_3_^–^, Cl^–^, and Br^–^) via significant, continuous cathodic shifts, as shown in Figure 6A.^64^

In all cases, a well-defined, monotonic, and reproducible response (Figures S28–S30) of the sensor was observed with the following response magnitude selectivity, defined by the maximum cathodic voltammetric shift Δ*E*_max_: H_2_PO_4_^–^ > Cl^–^ > Br^–^ > HSO_4_^–^ > NO_3_^–^, with an impressively large perturbation magnitude toward H_2_PO_4_^–^ of −217 mV (Table 3).^65−68^ Notably, as discussed above, ^1^H NMR anion titration investigations demonstrated that the neutral receptor **2.Te** displayed no detectable binding of most of these anions in acetone.

**Table 3:** Maximum Cathodic Voltammetric Shift Δ*E*_max_ (mV), Binding Constants to the Oxidized Receptor *K*_Ox_ = *K*_2.Te2+_ (M^–1^) and BEF of **2.Te** in the Presence of Various Anions in CH_3_CN, CH_3_CN/H_2_O 19:1, and CH_3_CN/H_2_O 9:1. *K*_Ox_ Was Obtained by Fitting of the Voltammetric Binding Isotherms According to Eq 1

	CH_3_CN			CH_3_CN/H_2_O 19:1			CH_3_CN/H_2_O 9:1		
anion	Δ*E*_max_^a^	*K*_2.Te2+_^b^	BEF^c^	Δ*E*_max_^a^	*K*_2.Te2+_^b^	BEF^c^	Δ*E*_max_^a^	*K*_2.Te2+_^b^	BEF^c^
Cl^–^	–157	10,200	458	–52	300	7.6	–30	88	3.2
Br^–^	–133	4050	180	–65	348	12.6	–42	141	5.2
H_2_PO_4_^–^	–217	174,000	4760	–81	902	23.6	–34	41	3.8
HSO_4_^–^	–63	333	11.7	–48	300	6.5	–19	n/a	n/a
NO_3_^–^	–44	127	5.6	–16	n/a	n/a	–18	n/a	n/a

a Estimated error ± 3 mV.

b In M^–1^, *K*_Red_ = *K*_2.Te_ is ≈ 0 in all cases.

c Binding enhancement factor (BEF = *K*_2.Te2+_/*K*_2.Te_), calculated from Δ*E*_max_ according to eq S2, see the Supporting Information for further details. n/a – not meaningful due to small response.

This confirms that, as expected, receptor oxidation to **2.Te^2+^** significantly enhances ChB potency and enables voltammetric anion sensing. Quantified analysis of the voltammetric response isotherms determined impressively large binding constants in the oxidized state of *K*_**2.Te2+**_ of up to 174,000 M^–1^ for H_2_PO_4_^–^ and for the halides with binding constants of 4050 and 10,200 M^–1^ for Br^–^ and Cl^–^, respectively (Table 3). The H_2_PO_4_^–^ preference in this nonaqueous solvent is, again, in good agreement with the aforementioned ^1^H NMR studies in which only this anion induced measurable, but weak binding to **2.Te** in d_6_-acetone.

The large-magnitude anion recognition-induced voltammetric perturbations of **2.Te** toward Cl^–^, Br^–^, and H_2_PO_4_^–^ correspond to an impressively substantial binding switch-ON upon oxidation with BEFs of 458, 180, and 4760, respectively (Table 3). This reversible ChB donor modulation, by up to over 3-orders of magnitude, is notably greater than that attainable by cooperative ion-pair recognition in related heteroditopic ChB receptors.^44^ We believe that this anion binding enhancement of **2.Te^2+^** is predominantly driven by specific ChB interactions (and not by electrostatics), as inferred from the comparisons of **1.Te^2+^**/**Se^2+^**/**H^2+^** (vide supra) as well as comparisons with similar bis(ferrocene-(iodo)triazole) XB and HB systems (vide infra).^53^ However, electrostatic interactions between the anion guest species and the receptor’s Fc^+^ groups may also play a role. The delineation of these contributions will be the subject of future studies.

**Figure 7:**
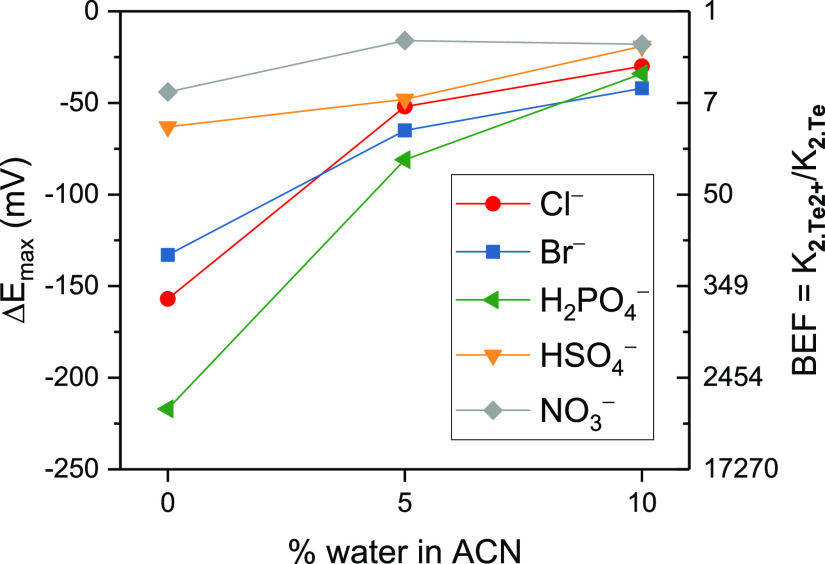
Maximum cathodic perturbations of **2.Te** toward a 50 mM concentration of various anions in CH_3_CN, CH_3_CN/H_2_O 19:1, and CH_3_CN/H_2_O 9:1, highlighting a change in selectivity and a significantly retained sensing capability in highly competitive aqueous solvents, in particular toward bromide. Also displayed is the magnitude of redox-induced binding switch-ON as quantified by the BEF according to eq S2. Connecting lines are to guide the eye only.

Unsurprisingly, the introduction of 5 or 10% water into the CH_3_CN solvent system is associated with moderately diminished response magnitudes, *K*_Ox_ binding constants, and BEFs (Figures 6B,C, 7, and Table 3).^49^ Nevertheless, significant shift responses and switching magnitudes are retained in these competitive, aqueous solvent systems, a particularly impressive accomplishment when considering that the native neutral **2.Te** receptor exhibits no significant anion affinity in less-competitive pure organic solvent media. For example, even in CH_3_CN/H_2_O 9:1, significant cathodic shifts of up to −41 mV toward Br^–^ are observed, corresponding to a BEF = 5.2.

Of further note, the response selectivity of the sensor is altered in the presence of water (Figure 7). The response toward the halides and dihydrogen phosphate, in particular, is attenuated upon introduction of 5% water, most likely a reflection of their respective large hydration enthalpies. This also results in a somewhat altered selectivity pattern in CH_3_CN/H_2_O 19:1 of Δ*E*_max_: H_2_PO_4_^–^ > Br^–^ > Cl^–^ ≈ HSO_4_^–^ > NO_3_^–^.

Upon increasing the water content to 10%, the response and binding magnitudes further decrease, however not as sharply. Again, the most strongly affected anion is H_2_PO_4_^–^, which now induces a similar response to Cl^–^, both of which are lower than that of Br^–^ (Br^–^ > H_2_PO_4_^–^ ≈ Cl^–^ > HSO_4_^–^ ≈ NO_3_^–^). The observation that the halide response is more significantly retained than that of the oxoanions hints at a unique halide selectivity associated with ChB recognition, akin to that observed in XB systems.^49^

### Chalcogen Bonding **1.Te^2+^** and **2.Te** Receptors as Potent Redox Controlled ON/OFF Switches and Sensors

In spite of their weak ChB anion binding strength in their reduced states and comparable structural simplicity, both **1.Te^2+^** and **2.Te** are among the most potent voltammetric anion sensors reported to date, as measured by their response magnitudes.^39^

In fact, to the best of our knowledge, **2.Te** significantly outcompetes every reported solution-phase XB voltammetric anion sensor, as well as the vast majority of redox-active HB anion sensors, for the sensing of most anions in CH_3_CN, in particular chloride and bromide.^33,39,46,48,49,60,69−72^ This is particularly noteworthy considering, in general, the significantly weaker sigma-hole donor ability of ChB receptors in comparison to XB systems.^18,19,44^ As shown in a comparison of the sensing performance of **2.Te** with related XB and HB bis(ferrocene-(iodo)triazole) systems (see SI Section 7 and Figures S31–S34),^49^ Br^–^ especially elicits an up to 2.4-fold larger response than its structurally closest XB congener, in both CH_3_CN and CH_3_CN/H_2_O 19:1. Due to its enhanced response magnitudes, **1.Te^2+^** even outcompetes these XB and HB sensors (recently reported as some of the most potent voltammetric sensors), for the sensing of Cl^–^ and Br^–^ at high concentrations in CH_3_CN/H_2_O 9:1.^49^ These observations support a developing picture that supports a ChB preference for the “softer” halides in comparison to oxoanions (akin to XB), in spite of their lower basicity.

As discussed above, the large response magnitudes of **1.Te^2+^** and **2.Te** are a direct reflection of their large anion binding enhancement factors (BEF = *K*_Ox_/*K*_Red_), that is the relative change in anion binding constants upon reduction/oxidation.^33,39^ The impressive sensing performance of both **1.Te^2+^** and **2.Te** can thus be attributed to a uniquely potent switching-OFF or -ON of anion binding, respectively, as previously observed in a range of XB voltammetric anion sensors. We ascribe this to the high sensitivity of the ChB donor strength to electronic inductive substituent effects^42^ as well as the most intimate spatial and electronic coupling of the sigma-hole donor binding site to the redox transducer, afforded by the direct appendage of the Te sigma-hole donor to Fc or viologen. As a result, the redox modulation of the electroactive transducer more strongly influences the ChB sigma-hole depth and thereby anion binding strength, which in turn translates to a large voltammetric response.

The direct appendage of Te ChB donor atoms with optical or redox transducer groups remains a largely unexplored, but promisingly highly potent, design feature of ChB receptors that not only enables sensing with improved sensitivity but can, in the case of the **2.Te** scaffold, also “free up” a substituent, such that other functional groups can easily be incorporated into the receptor (here via the triazole component). This is notably not easily achievable in XB or HB systems in which the XB/HB bond donor atom typically carries only one substituent.

As a result of this structural adaptability, combined with the high-fidelity redox-control of ChB potency, as demonstrated with **1.Te^2+^** and **2.Te**, we envision these electroactive ChB centers as ideal motifs for further exploration in ion sensors as well as molecular redox switches and machines.

## Conclusions

We report, for the first time, ChB-mediated electrochemical anion sensing and reversible redox-controlled switch-ON and switch-OFF of ChB donor potency exemplified by 3,5-bis-ferrocenetellurotriazole pyridine and telluroviologen anion receptors. Despite their structural simplicity and comparably weak anion binding strength in the reduced receptor states, both systems displayed large scale anion binding enhancements, of up to 3 orders of magnitude, in the oxidized state. This is directly reflected in large cathodic voltammetric shifts in the presence of anions, which persist even in the competitive CH_3_CN/H_2_O 9:1 solvent system, with a noteworthy selectivity toward the halides, in particular bromide. Impressively, the high sensitivity of these sensors compares very favorably with related XB and HB sensors, in some cases outcompeting the best XB sensors reported to date.^49^ This high sensitivity can be attributed to a unique, most intimate electronic coupling of redox-active center and ChB donor sites and the sensitivity of sigma hole donors to inductive effects. These observations lend further support to recent findings that ChB interactions are highly electronically tunable^42^ and not only pave the way toward the further exploitation of ChB in anion sensor design and redox switches but also present fundamental insights into the nature of ChB interactions.

## Supporting Information Available

Experimental details and materials, additional data, and figures (PDF)
