# Supplementary material for: Redox-Switchable Chalcogen Bonding for Anion Recognition and Sensing
Source: J Am Chem Soc. 2022 May 6;144(19):8827–36. doi: 10.1021/jacs.2c02924 (PMC9121379; doi:10.1021/jacs.2c02924)
Supplement: Supplementary file 1 — ja2c02924_si_001.pdf [file ja2c02924_si_001.pdf]

Supporting Information

Redox-Switchable Chalcogen Bonding for Anion Recognition and Sensing

Robert Hein, Andrew Docker, Jason J. Davis, Paul D. Beer**

Department of Chemistry, University of Oxford, South Parks Road, Oxford OX1 3QZ, U. K.

**jason.davis@chem.ox.ac.uk, paul.beer@chem.ox.ac.uk*

Table of Contents

1. Experimental 2
2. Synthesis 4
3. ^{1}H NMR and UV-vis Anion Binding Studies 16
4. Voltammetric Characterisation of Receptors 19
5. Voltammetric Anion Binding Studies 25
6. Determination of Binding Enhancement Factors (BEFs) 28
7. Receptor Comparisons 29
8. References 32

1. Experimental

General Information

All commercially available chemicals and solvents were used as received without further purification. All hygroscopic TBA salts were stored in vacuum desiccators at room temperature. Dry solvents were degassed with N_2 and dried on a Mbraun MPSP-800 column. Ultrapure water was obtained from a Milli-Q system (18.2 MΩcm). Mass spectrometry was performed on a Bruker micrOTOF. NMR spectra were recorded on Bruker NMR spectrometers (AVIII HD 500 or AVIII HD 400). Chromatography was performed using silica gel (particle size: 40-63 µm). All data analysis and fitting was carried out with OriginPro 2017.

^{1}H NMR Titrations

In a typical titration experiment, the host solution (1.0 mM, 0.5 mL) was titrated with anion (as TBA salt) which was dissolved in the same deuterated solvent. Data fitting was carried according to a 1:1 stoichiometric host-guest binding model.[1]

Electrochemical Measurements

All experiments were conducted using an Autolab Potentiostat (Metrohm) in a three-electrode setup using a glassy carbon (GC) working electrode (3 mm, BASi), a Pt wire counter electrode and a Ag|$AgNO_3$ reference electrode (with an inner filling solution of 10 mM $AgNO_3$, 100 mM $TBAClO_4$ in ACN). Unless otherwise noted, 100 mM $TBAPF_6$ was used as supporting electrolyte. CVs were recorded using a step potential of 2.4 mV and at a scan rate of 100 mV/s, unless otherwise noted. The electrochemical reversibility was assessed by recording CVs at varying scan rates (25, 50, 75, 100, 200, 400, 600 and 800 mV/s). SWVs were recorded using a step potential of 2 mV, a 20 mV amplitude and at a frequency of 25 Hz. All electrochemical titrations were followed by SWV. The receptors` half-wave potentials were determined as the peak potential by SWV and were referenced to Fc as an internal standard in all cases apart from the measurements in DCM/$TBABAr^F_4$ where Fc was used as an external standard in

$DCM/TBAPF_6$. All electrochemical studies on the viologen receptors **1.Te^{2+}/Se^{2+}/H^{2+}** were carried out in argon-degassed solutions.

Voltammetric titration experiments were carried at a constant receptor concentration of 0.1 mM. In all cases the overall ion concentration was kept constant by titration of an initial host solution containing 100 mM $TBAPF_6$ with an anion solution in the same solvent system containing 100 mM TBA-anion. Prior to each titration the GC electrode was cleaned by mechanical polishing with an alumina slurry (0.05 µm particle size), followed by sonication in H_2O/EtOH. In some cases the appearance of broad shoulder peaks was observed during the course of the titration of **2.Te** in ACN. In these cases the working electrode was periodically repolished resulting in the "recovery" of the expected voltammograms (without shoulder peaks). This crucially confirms that these peaks arise from physisorption of an *in situ* generated species and not from the receptor itself. Voltammetric shifts were determined by SWV. ΔE_{max} was assessed at the highest measured anion concentration (50 mM) and refers to the most cathodic peak in the case where more than one peak is observed (in the case of **2.Te** with halides in ACN).

UV-vis Measurements

All UV-vis studies were carried out on a HORIBA Duetta spectrometer. Titration experiments were carried out in 10 x 10 mm cuvettes on a 0.1 mM host solution, which was titrated with a 10 mM anion solution containing 0.1 mM host.

2. Synthesis

Synthesis of 1.Te^{2+}/Se^{2+}/H^{2+}

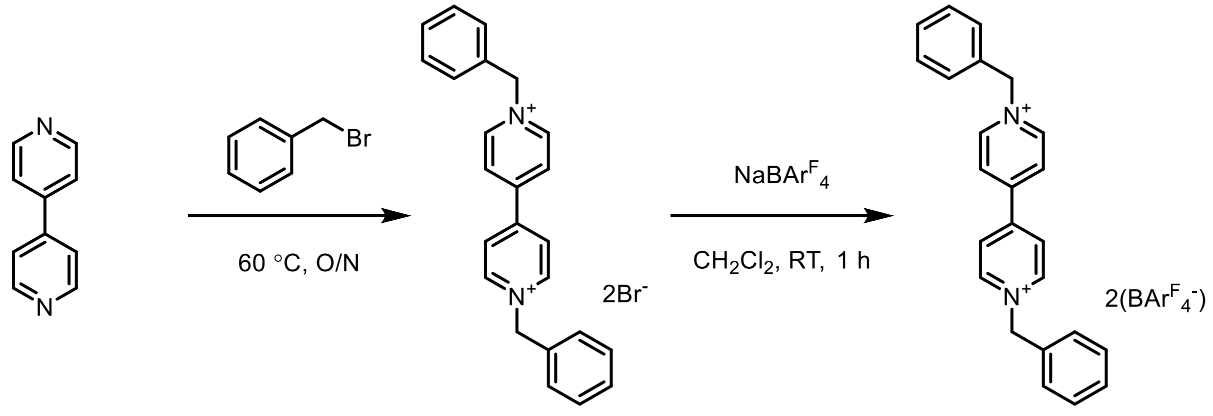

Scheme 1. Synthesis of **1.H(BAr^F$_4$)$_2$.**

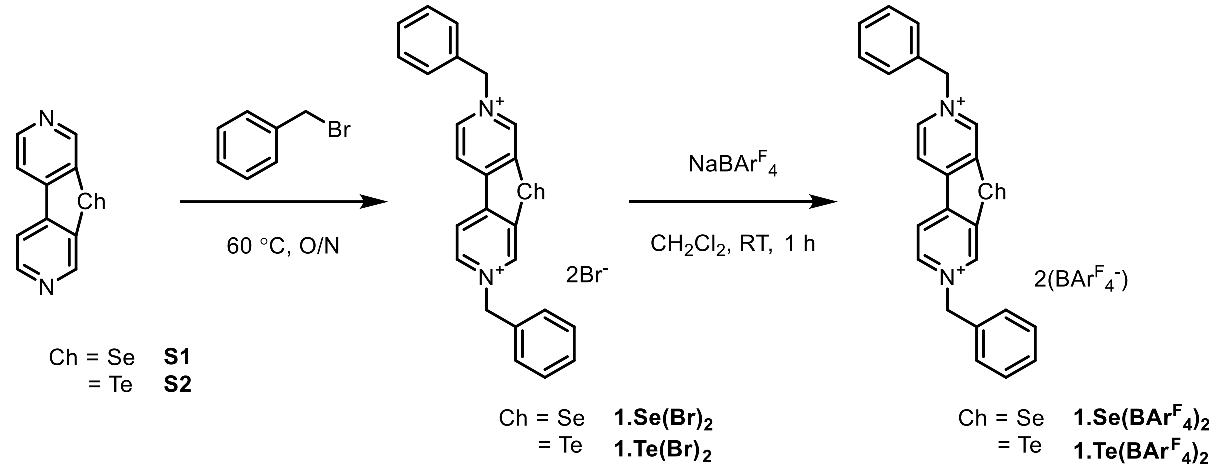

Scheme 2. Synthesis of **1.Se(BAr^F$_4$)$_2$** and **1.Te(BAr^F$_4$)$_2$.**

S1 and **S2** were synthesised according to the previously reported procedure by He and co-workers.[2]

The synthesis of **1.H(BAr^F$_4$)$_2$**, **1.Se(BAr^F$_4$)$_2$** and **1.Te(BAr^F$_4$)$_2$** was conducted according the following general procedure:

The appropriate 4,4′-bipyridine derivative (0.25 mmol) was dissolved in neat benzyl bromide (5 mL) and heated to 60 °C in a sealed microwave vial overnight. After cooling to room temperature, the formed precipitate was isolated by vacuum filtration and washed copiously with CH_2Cl_2 (250 mL) to afford the corresponding dibromide salt. The bipyridinium dibromide

was subsequently suspended in CH_2Cl_2 (3 mL) to which was added $NaBAr^F{}_4$ (2 eq.) and left to stir for 30 minutes at room temperature, after which time the mixture was diluted with CH_2Cl_2 (100 mL) and washed with H_2O (3 x 50 mL). The organic solvent was removed in vacuo to afford the corresponding bipyridinium as its $(BAr^F{}_4{}^-)_2$ salt.

1.H(BAr$^F{}_4$)$_2$

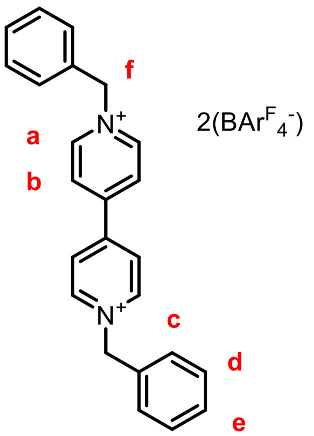

Isolated as a white solid (419 mg, 0.203 mmol, 81 %).

^{1}H NMR (500 MHz, CD_3CN) δ 8.95 (d, *J* = 6.5 Hz, $4H_a$), 8.33 (d, *J* = 6.5 Hz, $4H_b$), 7.78 – 7.63 (m, $24H_{BArF}$), 7.55 – 7.45 (m, $10H_{c,d,e}$), 5.81 (s, $4H_f$).

^{19}F NMR (470 MHz, CD_3CN) δ -63.25.

^{13}C NMR (126 MHz, CD_3CN) δ 146.58, 135.66, 133.53, 131.18, 130.64, 130.32, 129.96 (d), 128.50, 126.55, 124.39, 122.23, 65.78. Signals missing due to poor solubility and C-F and C-B coupling.#

HRMS (ESI+ve) m/z: 338.1779 ($[M-2(BAr^F{}_4)]^{\cdot+}$, $C_{24}H_{20}N_2$ requires 338.1778).

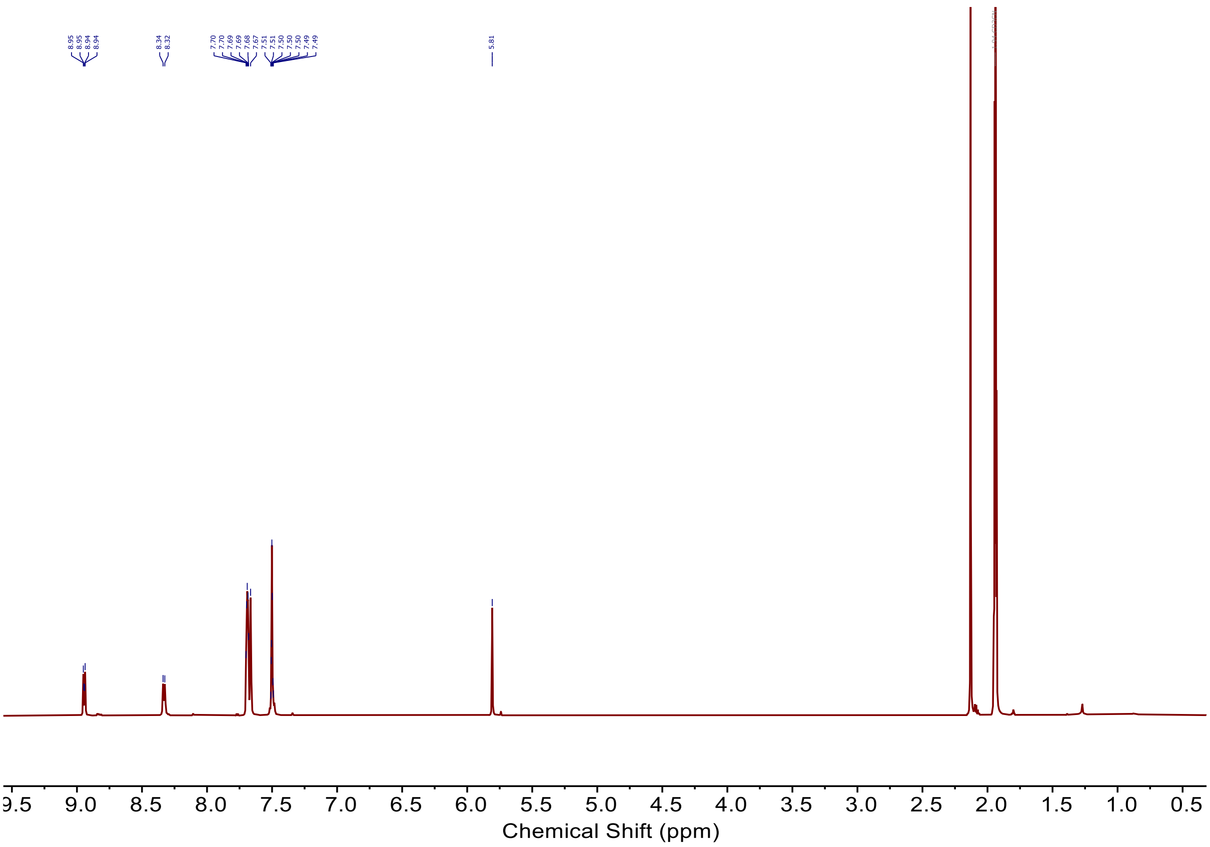

Figure S1. ^{1}H NMR spectrum of **1.H(BAr$^{F}_{4}$)$_2$** (CD_3CN, 500 MHz, 298 K).

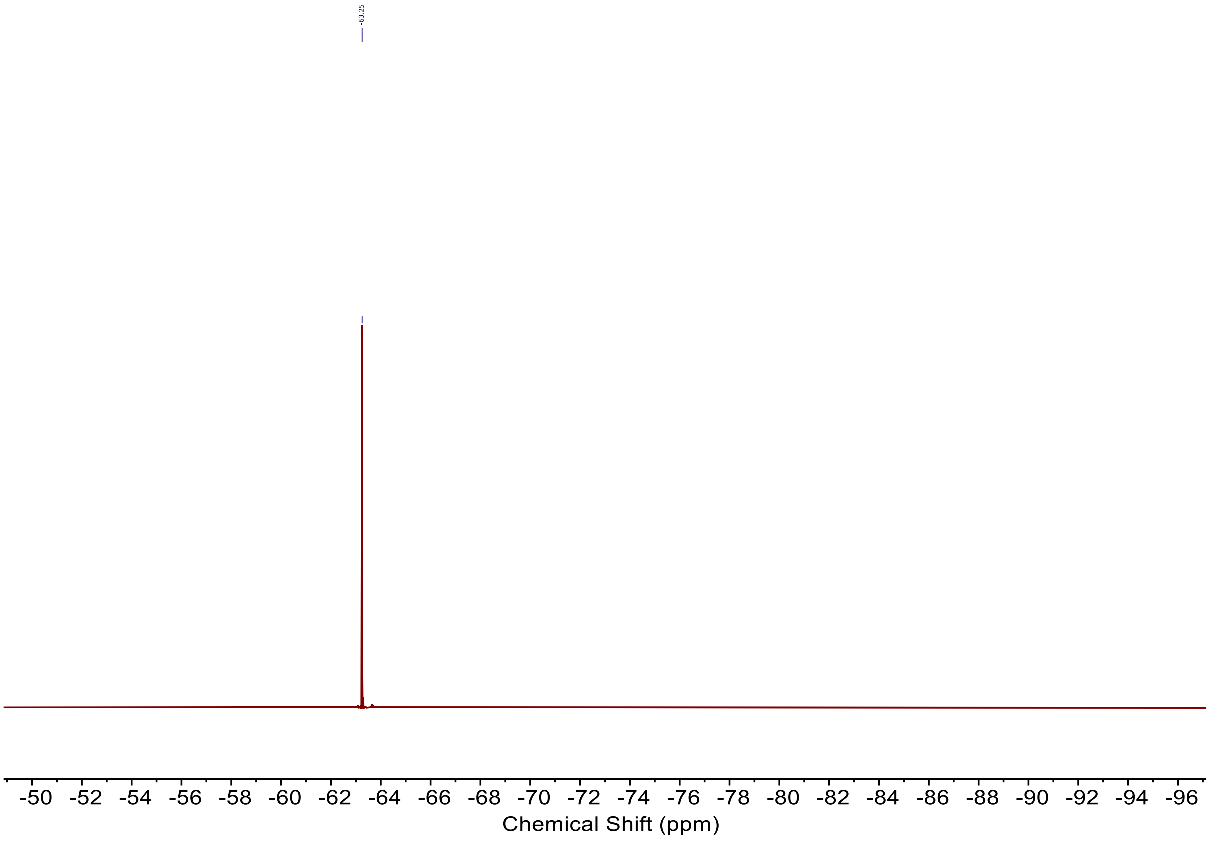

Figure S2. ^{19}F NMR spectrum of **1.H(BAr$^{F}_{4}$)$_2$** (CD_3CN, 470 MHz, 298 K).

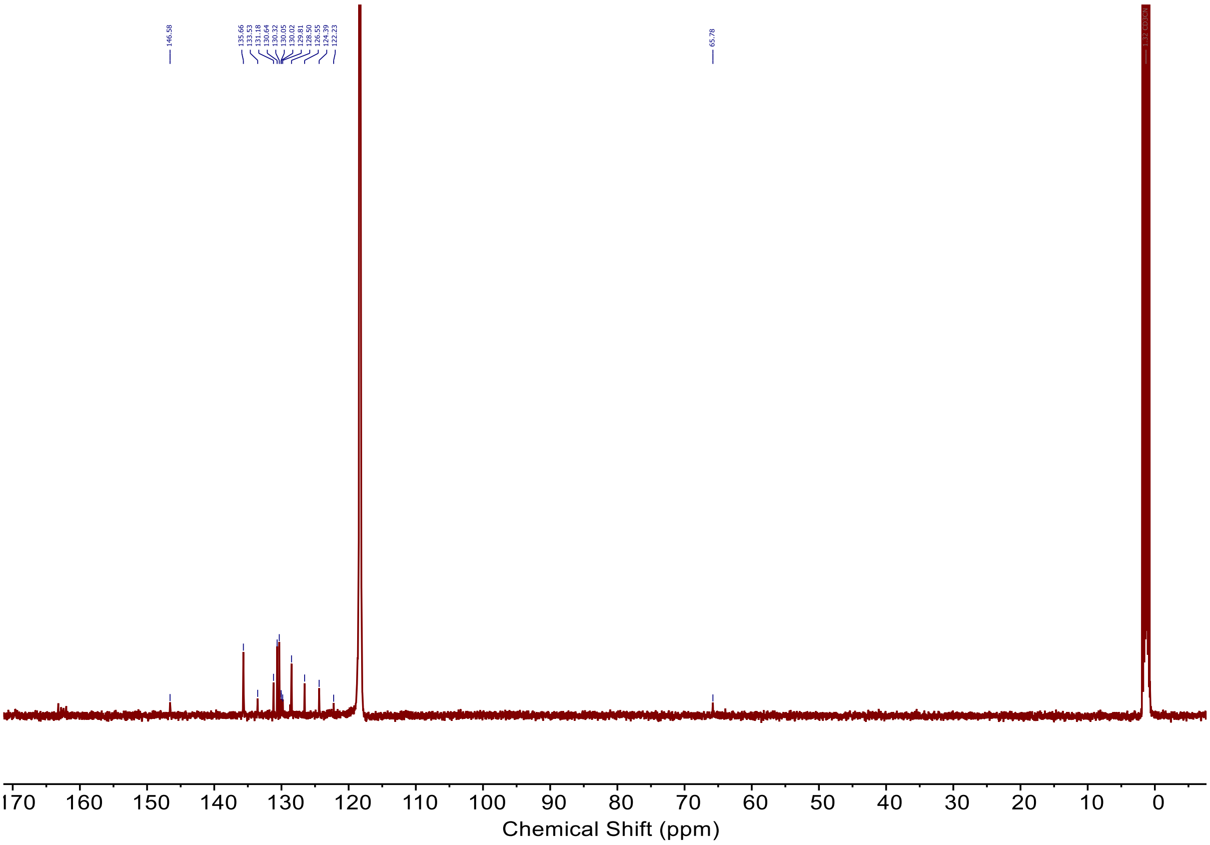

Figure S3. ^{13}C NMR spectrum of **1.H(BAr^F$_4$)$_2$** (CD_3CN, 126 MHz, 298 K).

1.Se(BAr^F$_4$)$_2$

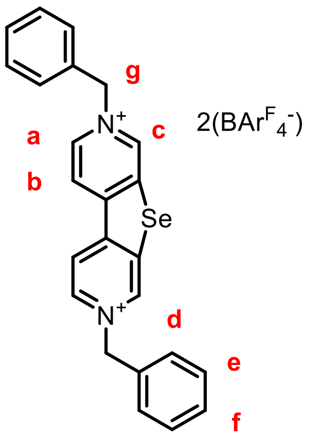

Isolated as a yellow green solid (306 mg, 0.143 mmol, 57 %).

^{1}H NMR (500 MHz, CD_3CN) δ 9.69 (s, $2H_c$), 9.01 (s, $4H_{a,b}$), 7.77 – 7.59 (m, $24H_{BArF}$), 7.59 – 7.41 (m, $10H_{d,e,f}$), 5.94 (s, $4H_g$).

^{19}F NMR (470 MHz, CD_3CN) δ -63.25.

^{13}C NMR (126 MHz, CD_3CN) δ 162.63 (q, 49.9 Hz), 147.28, 145.66, 144.75, 141.17, 135.68, 133.18, 131.33, 130.88, 130.69, 129.95 (qdd, *J* = 31.7, 5.8, 2.9 Hz), 128.73, 126.57, 125.70, 124.41, 122.24, 118.69 (q, *J* = 4.1 Hz), 66.55.

HRMS (ESI+ve) m/z: 416.0788 ($[M-2(BAr^F{}_4)]^{\cdot+}$, $C_{24}H_{20}N_2Se$ requires 416.0786).

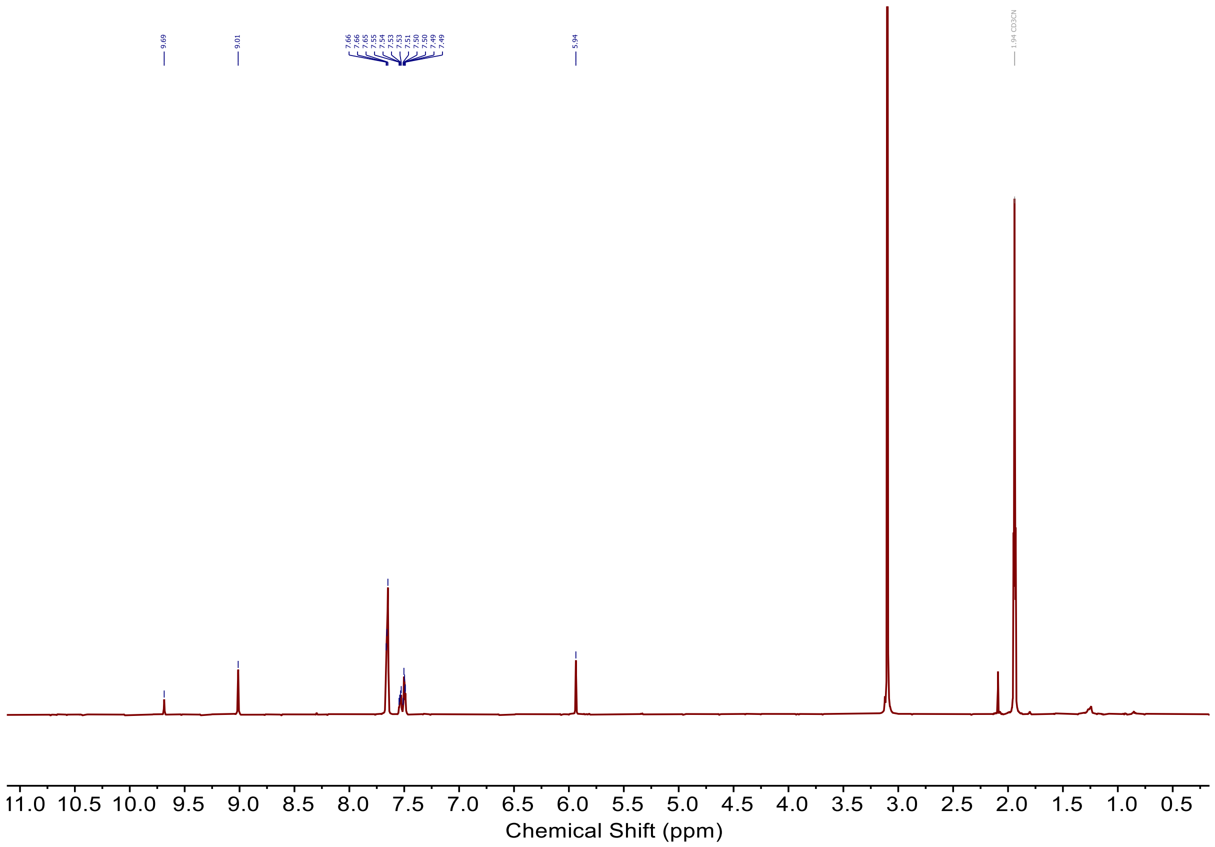

Figure S4. ^{1}H NMR spectrum of **1.Se(BAr$^F{}_4$)$_2$** (CD_3CN, 500 MHz, 298 K).

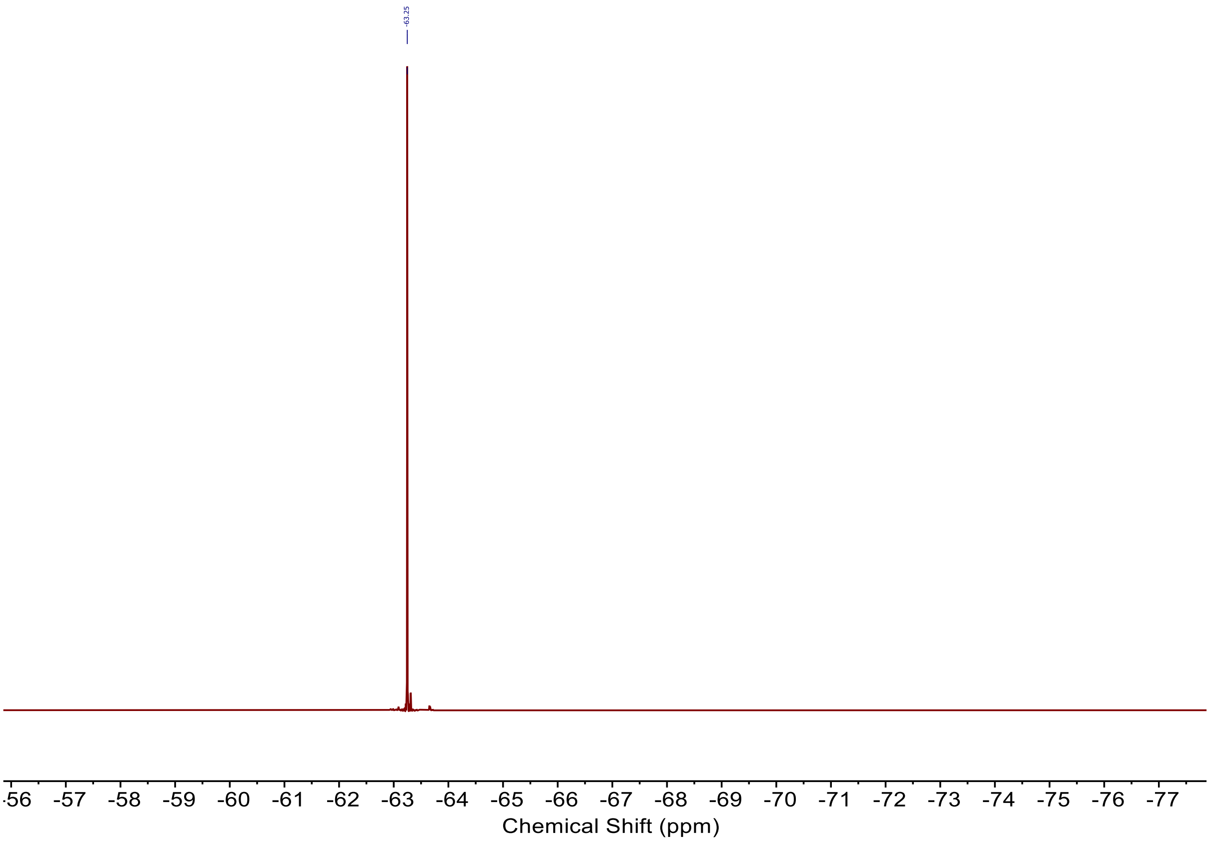

Figure S5. ^{19}F NMR spectrum of **1.Se(BAr^F$_4$)$_2$** (CD_3CN, 470 MHz, 298 K).

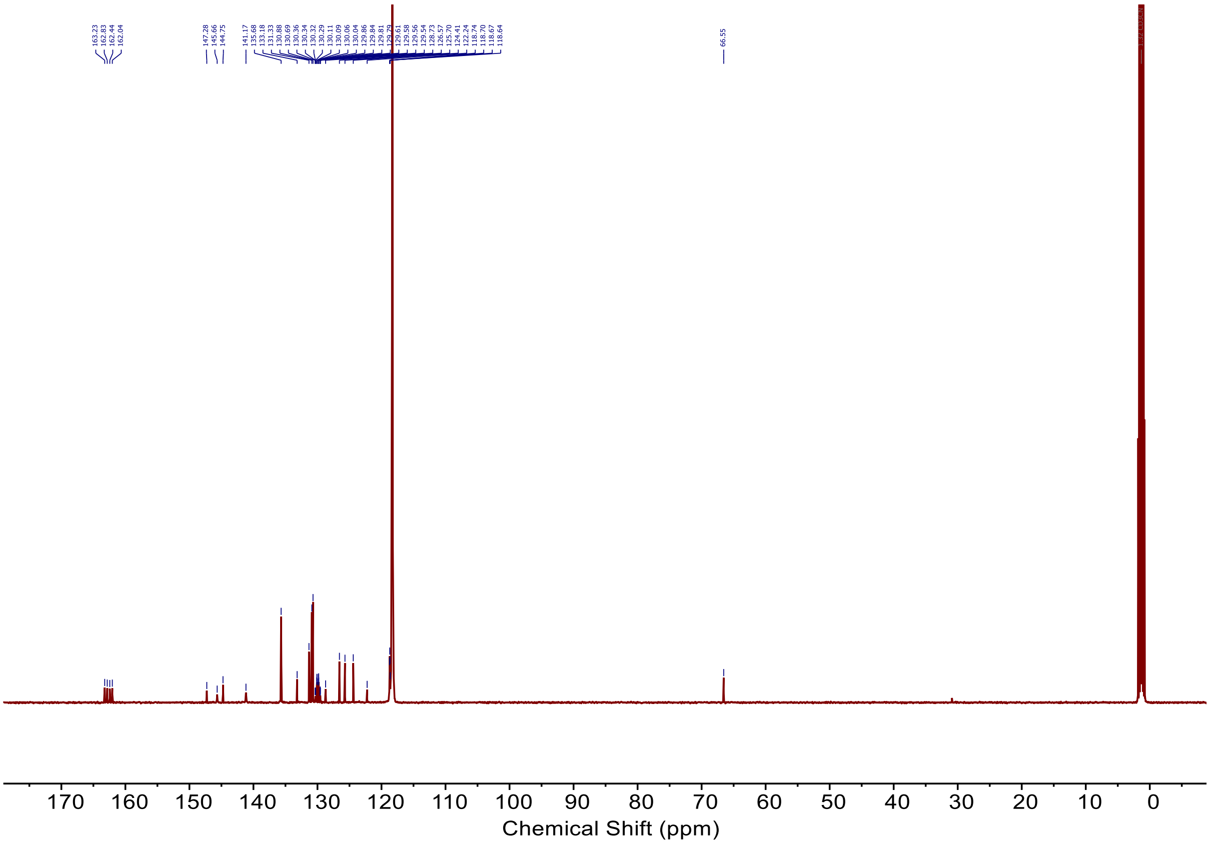

Figure S6. ^{13}C NMR spectrum of **1.Se(BAr^F$_4$)$_2$** (CD_3CN, 126 MHz, 298 K).

1.Te(BAr^F$_4$)$_2$

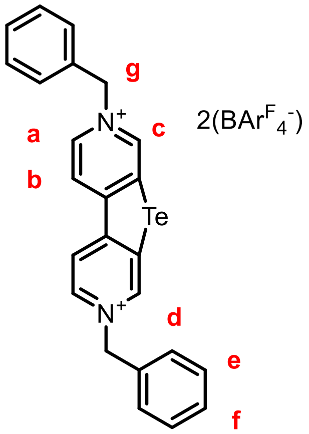

Isolated as a red solid (335 mg, 0.153 mmol, 61 %).

^{1}H NMR (500 MHz, CD_3CN) δ 9.56 (s, 2H_c), 8.95 – 8.84 (m, 4$H_{a,b}$), 7.74 – 7.60 (m, 24H_{BArF}), 7.53 (h, *J* = 3.8, 3.1 Hz, 10$H_{d,e,f}$), 5.86 (s, 4H_g).

^{19}F NMR (470 MHz, CD_3CN) δ -63.25.

^{13}C NMR (126 MHz, CD_3CN) δ 162.63 (q, 49.9 Hz), 147.28, 145.66, 144.75, 141.16, 135.67, 133.17, 131.32, 130.87, 130.69, 129.95 (qdd, *J* = 31.7, 5.8, 2.9 Hz), 128.72, 126.56, 125.70, 124.40, 122.24, 118.68 (q, *J* = 4.1 Hz), 66.55.

HRMS (ESI+ve) m/z: 233.0338 ([M-2(BAr^F$_4$)]$^{2+}$, $C_{24}H_{20}N_2Te$ requires 233.0339).

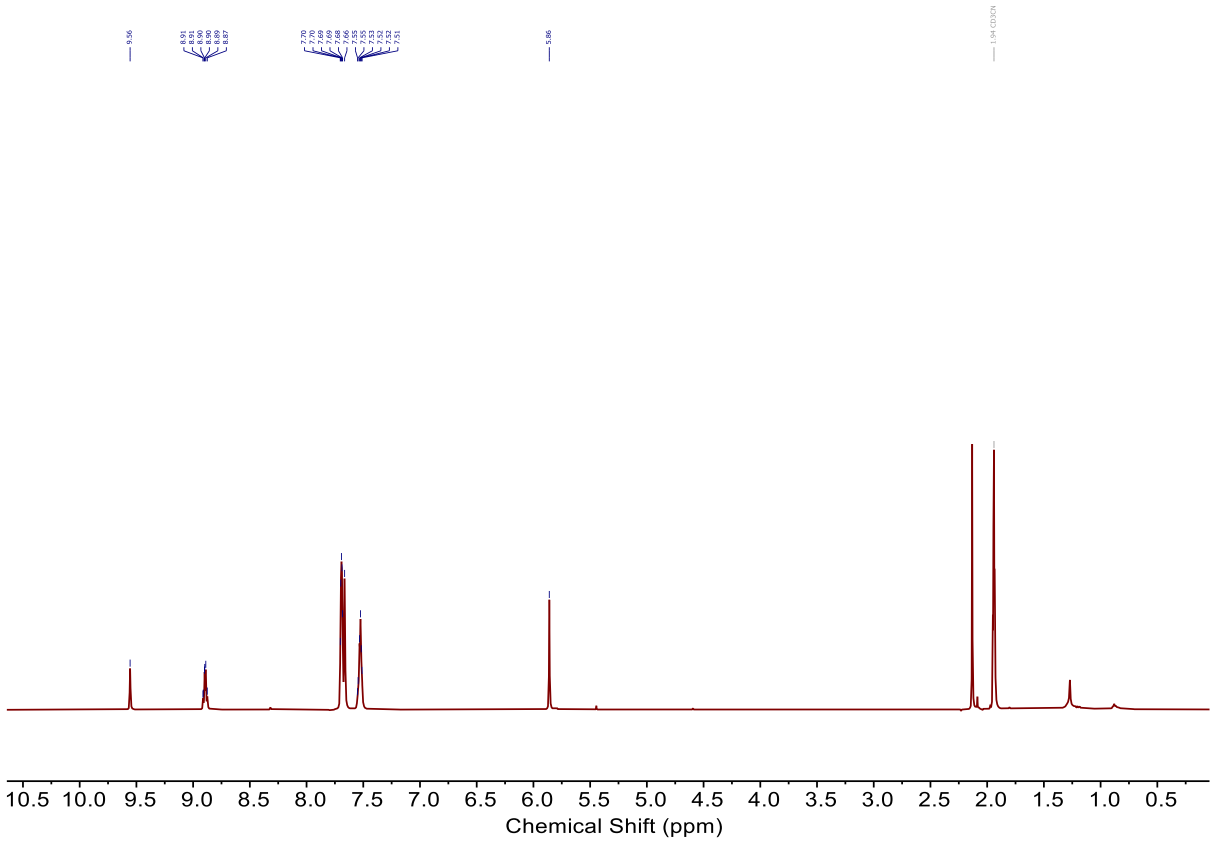

Figure S7. ^{1}H NMR spectrum of **1.Te(BAr$^{F}_{4}$)$_2$** (CD_3CN, 500 MHz, 298 K).

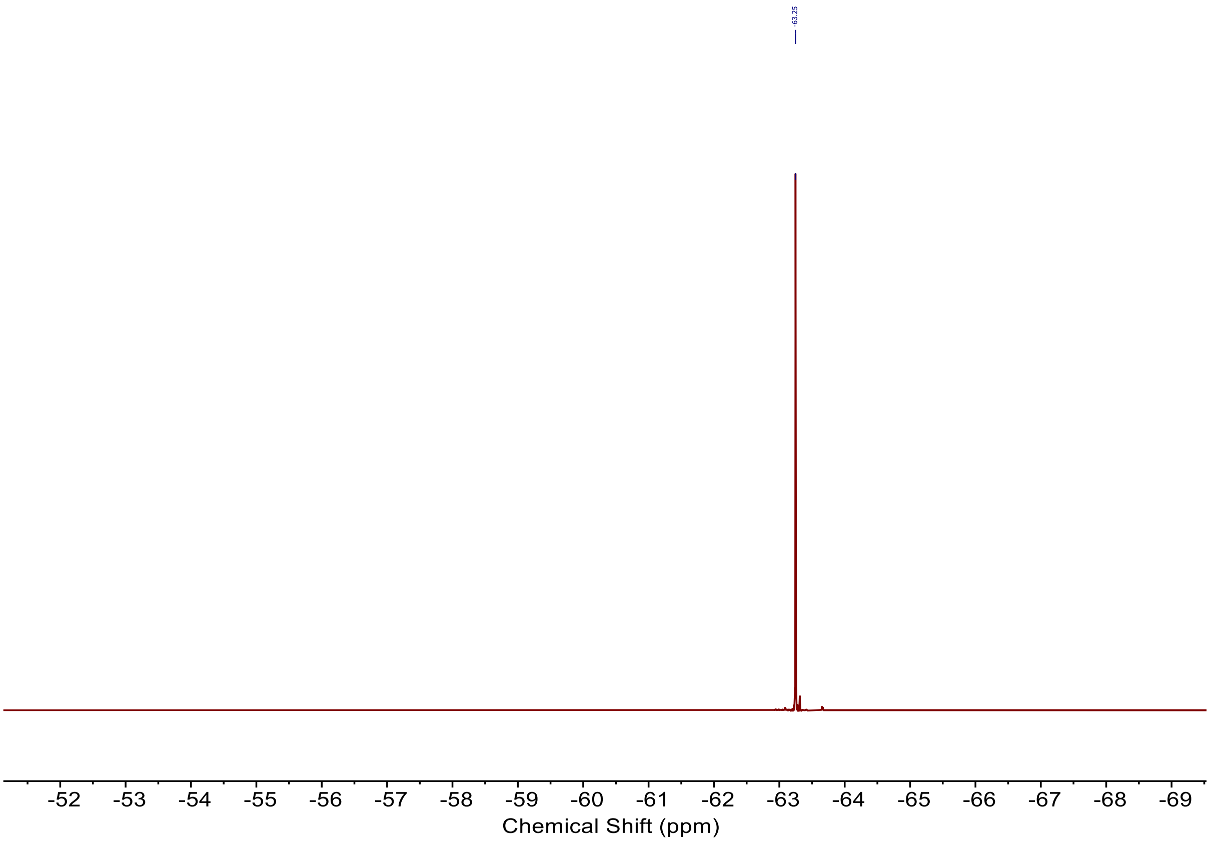

Figure S8. ^{19}F NMR spectrum of **1.Te(BAr^F$_4$)$_2$** (CD_3CN, 470 MHz, 298 K).

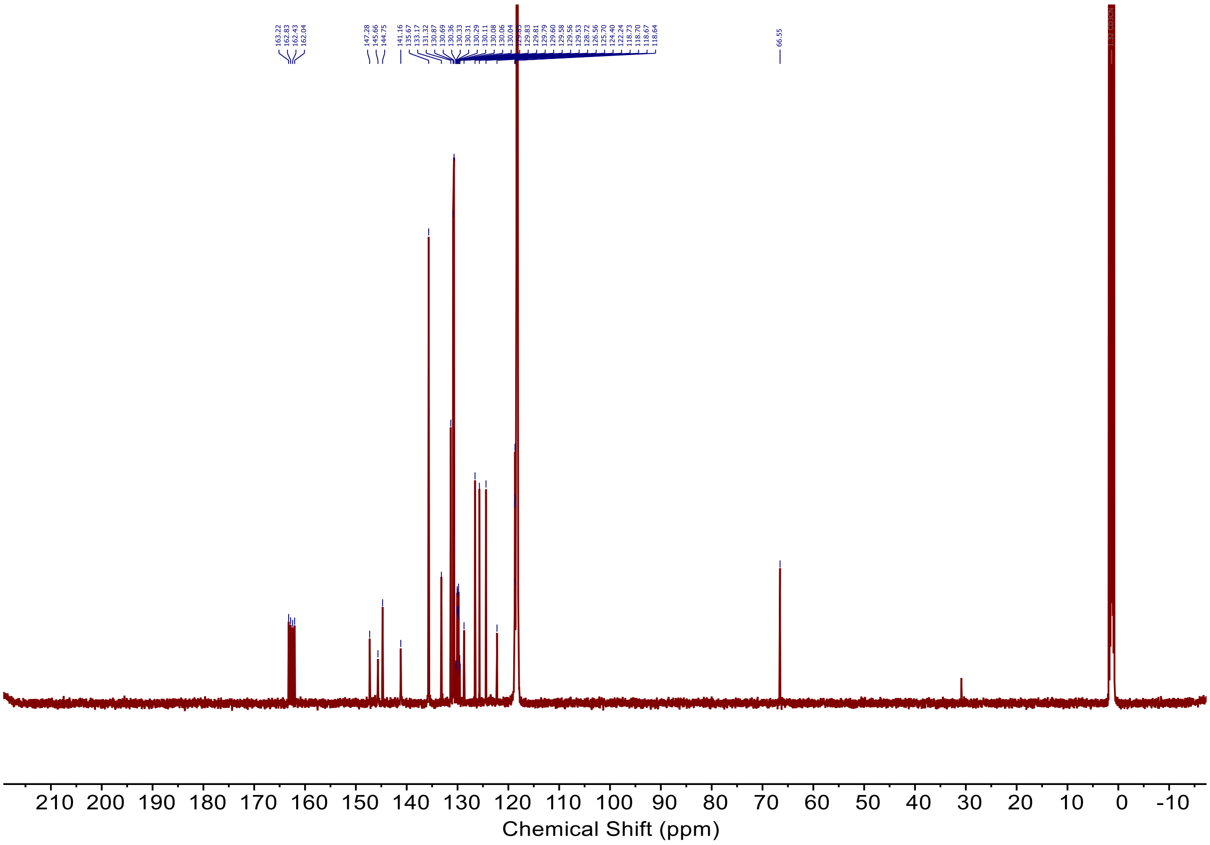

Figure S9. ^{13}C NMR spectrum of **1.Te(BAr^F$_4$)$_2$** (CD_3CN, 126 MHz, 298 K).

Synthesis of 2.Te

Azido pentafluorobenzene[3], diferrocenyl ditelluride[4] and bis(silveracetylide) **3**[5] were synthesised according to literature procedures.

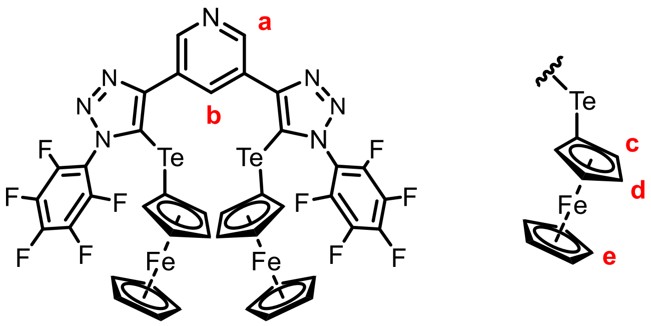

3 (150 mg, 0.440 mmol) was suspended in anhydrous THF (20 mL) under an atmosphere of nitrogen and cooled to 0 °C. In a separate flask diferrocenyl ditelluride (276 mg, 0.444 mmol) was dissolved in a THF (2 mL) and CH_2Cl_2 (2 mL) mixture and cooled to 0 °C. To the diferrocenyl ditelluride solution a 1 M Br_2 solution in CH_2Cl_2 (0.444 mL) was added dropwise, and left to stir for 2 min, after which the mixture was added dropwise to the THF suspension of **3**. The mixture was allowed to warm to room temperature and stirred for 30 minutes protected from light. The mixture was filtered through celite, diluted with CH_2Cl_2 (ca. 250 mL) and washed with water (2 x 50 mL), the organic layer was dried over $MgSO_4$ and concentrated to dryness in vacuo. The crude material was rapidly passed through a silica plug eluting with $CHCl_3$ (ca. 250 mL) and concentrated to dryness in vacuo to afford the crude bis-ferrocenyl-tellurium appended alkyne **4** as an orange solid.

In a separate flask $[Cu(MeCN)_4]PF_6$ (33 mg, 0.088 mmol) and TBTA (45 mg, 0.088 mmol) were dissolved in an anhydrous degassed THF (2 mL) and CH_2Cl_2 (2 mL) mixture and left to stir for 10 min under an atmosphere of nitrogen. After which time the crude bis-ferrocenyl-tellurium appended alkyne **4** was added as a solid followed by azido pentafluorobenzene (460 mg, 2.20 mmol) and left to stir at room temperature until determined complete by TLC analysis (ca. 4 h). The reaction mixture was diluted with CH_2Cl_2 (100 ml) and washed with a 0.1 M EDTA/NH_4OH (25 mL) solution, the organic phase was collected, dried over $MgSO_4$ and concentrated to dryness. The crude mixture was subjected to purification by column

chromatography, eluting with CH_2Cl_2:EtOAc mixtures to afford **2.Te** as an orange solid (288 mg, 0.246 mmol, 56% over two steps).

^{1}H NMR (500 MHz, $CDCl_3$) δ 9.30 (s, 2H_a), 8.92 (s, 1H_b), 4.23 (t, *J* = 1.8 Hz, 4H_c), 4.18 (s, 10H_e), 4.13 (t, *J* = 1.8 Hz, 4H_d).

^{13}C NMR (126 MHz, $CDCl_3$) δ 149.60, 148.13,143.89 (dm, *J* = 260 Hz), 143.20 (dm, *J* = 260 Hz) 137.98 (dm, *J* = 260 Hz), 135.44, 127.25, 113.68 (d, *J* = 17.1 Hz), 108.11, 79.48, 72.81, 69.55, 43.65.

HRMS (ESI+ve) m/z: 1171.9549 ($[M+H]^+$, $C_{41}H_{21}F_{10}Fe_2N_7Te_2$ requires 1171.8577).

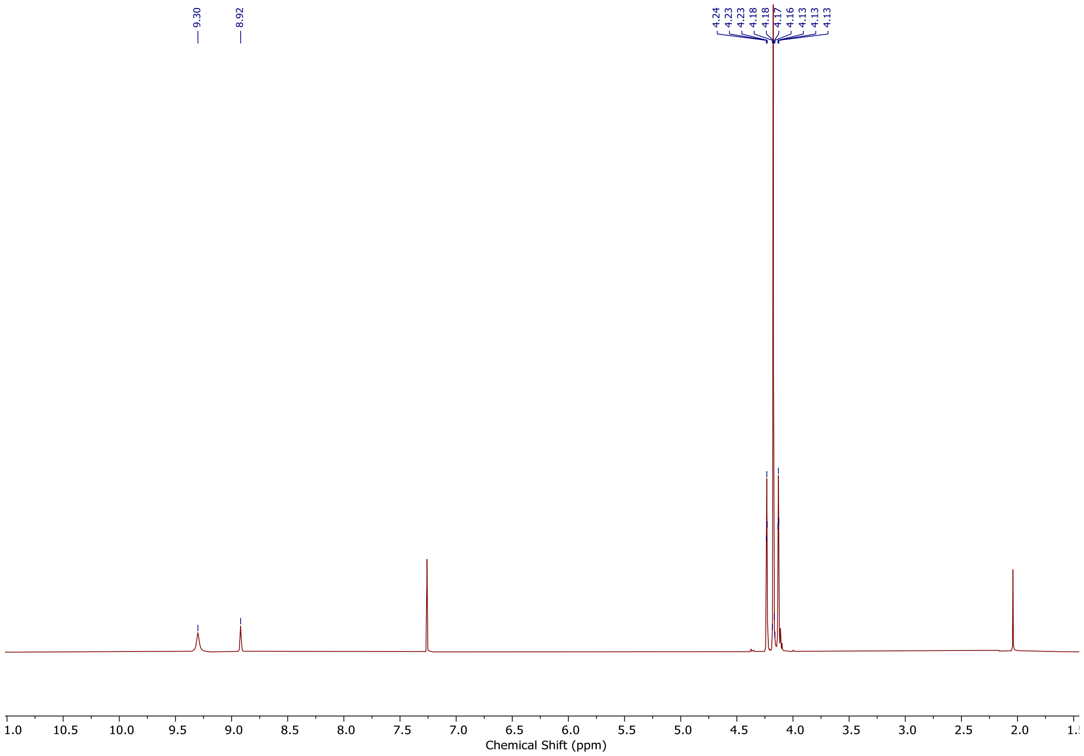

Figure S10. ^{1}H NMR Spectrum of **2.Te** (500 MHz, $CDCl_3$).

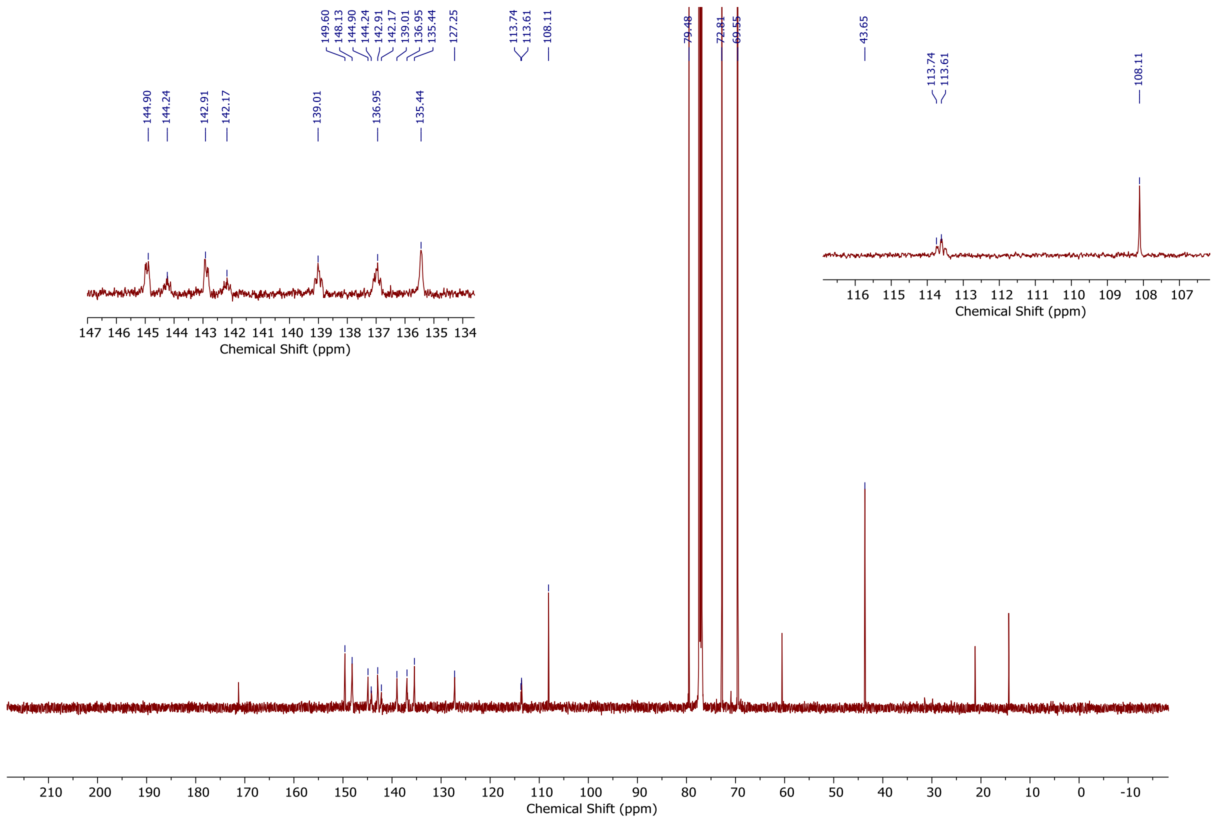

Figure S11. ^{13}C NMR Spectrum of **2.Te** (125 MHz, $CDCl_3$).

3. ^{1}H NMR and UV-vis Anion Binding Studies

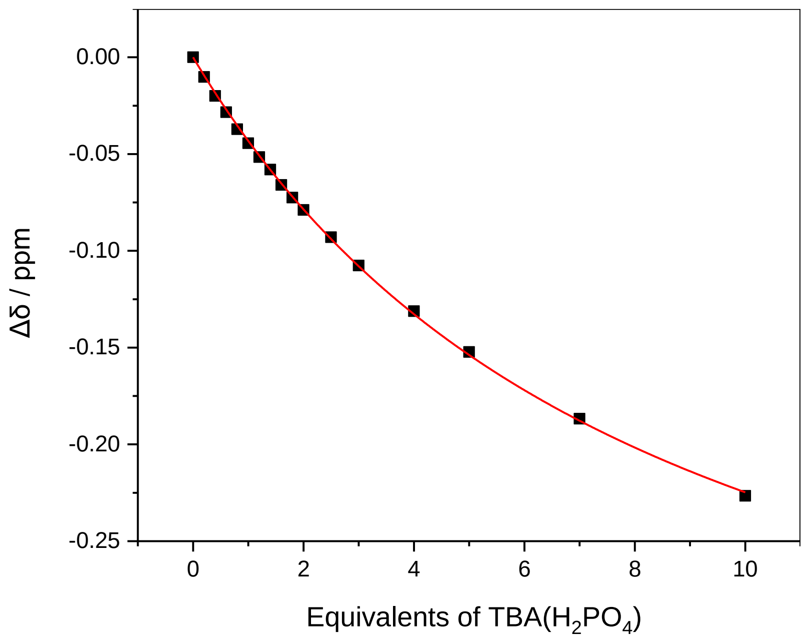

Figure S12. Chemical shift perturbation of ferrocene proton *c* of **2.Te** upon addition of increasing amounts of $H_2PO_4^-$ in d_6-acetone. Anion titration binding curve for **2.Te**. The solid red line represents a fit according to 1:1 host-guest stoichiometric binding.

Table S1. Anion association constants K_a of neutral **2.Te** toward various anions as determined by ^{1}H NMR titrations in acetone-d_6. Anions were added as their tetrabutylammonium salts and K was determined by analysis of ferrocene proton *c*. / - no binding

**Anion**	**$H_2PO_4^-$**	**NO_3^-**	**Cl^-**	**Br^-**	**I^-**
**K_a (M^{-1})**	111 ± 11	/	/	/	/

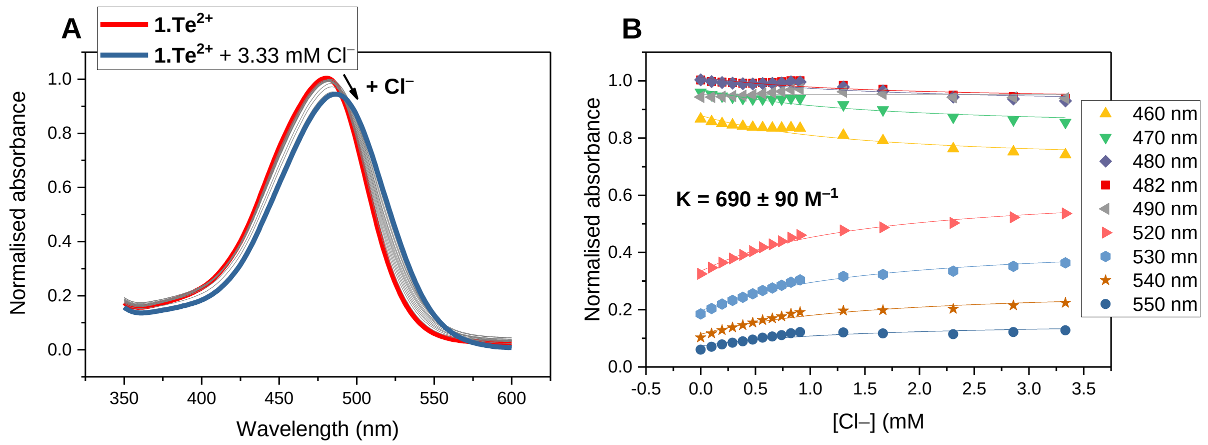

Figure S13. A) Normalised UV-vis spectra of 100 µM **1.Te^{2+}** in ACN/H_2O 9:1 in the absence (red line) and presence (blue line) of 3.33 mM Cl^-. B) The corresponding binding isotherms at selected wavelengths fitted by global fitting to the 1:1 host-guest stoichiometric binding model.

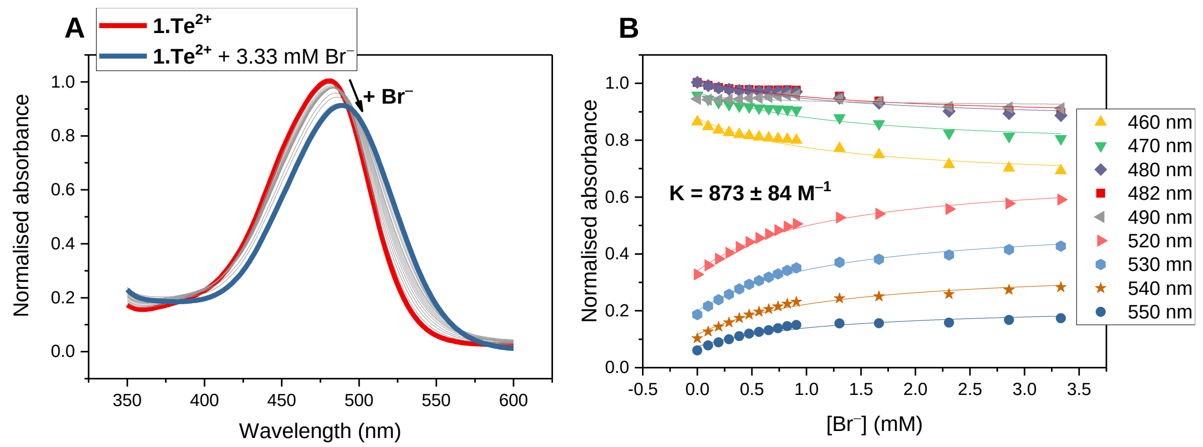

Figure S14. A) Normalised UV-vis spectra of 100 µM **1.Te^{2+}** in ACN/H_2O 9:1 in the absence (red line) and presence (blue line) of 3.33 mM Br^-. B) The corresponding binding isotherms at selected wavelengths fitted by global fitting to the 1:1 host-guest stoichiometric binding model.

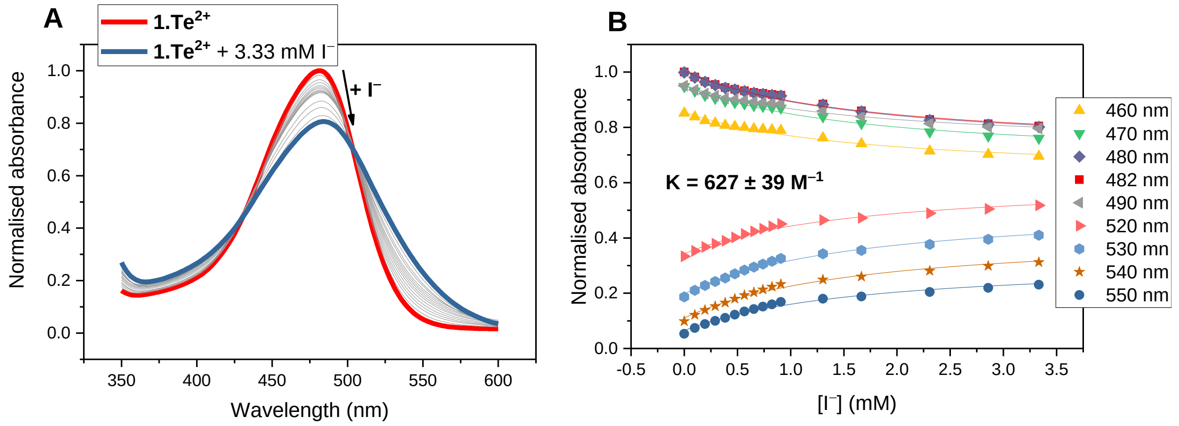

Figure S15. A) Normalised UV-vis spectra of 100 μM **1.Te^{2+}** in ACN/H_2O 9:1 in the absence (red line) and presence (blue line) of 3.33 mM I$^-$. B) The corresponding binding isotherms at selected wavelengths fitted by global fitting to the 1:1 host-guest stoichiometric binding model.

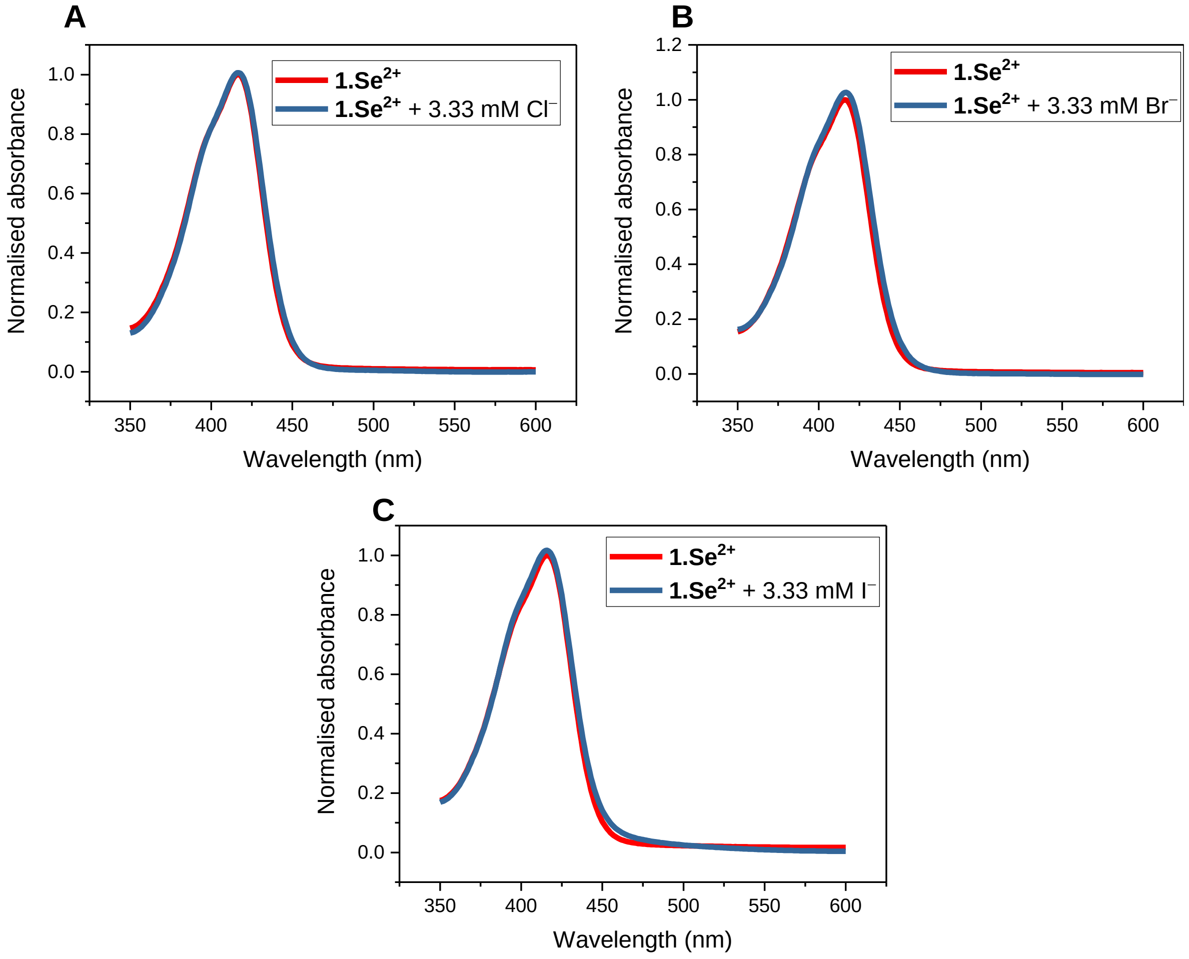

Figure S16. Normalised UV-vis spectra of 100 μM **1.Se^{2+}** in ACN/H_2O 9:1 in the absence (red line) and presence (blue line) of 3.33 mM halide anions. A) Cl$^-$. B) Br$^-$ and C) I$^-$.

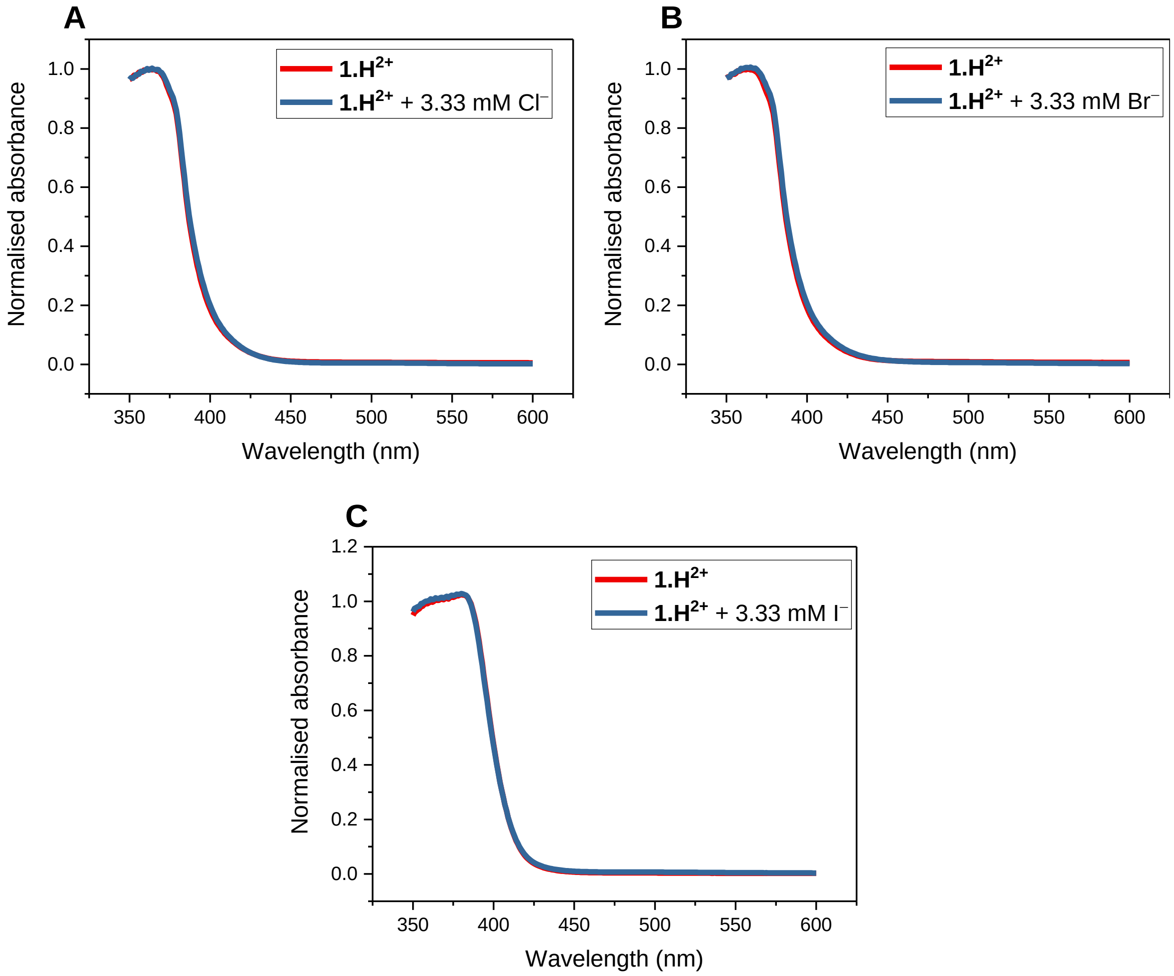

Figure S17. Normalised UV-vis spectra of 100 µM **1.H^{2+}** in ACN/H_2O 9:1 in the absence (red line) and presence (blue line) of 3.33 mM halide anions. A) Cl^-. B) Br^- and C) I^-.

4. Voltammetric Characterisation of Receptors

Table S2. Half-wave potentials ($E_{1/2}$ (V) vs Fc/Fc$^+$) of both reductive couples of **1.Te^{2+}/Se^{2+}/H^{2+}** in degassed ACN, H_2O 9:1, 100 mM $TBAPF_6$ as determined by SWV. Values in parentheses represent the CV peak separation (in mV) at ν = 100 mV/s. Estimated error ≤5 mV.

ACN/H_2O 9:1	$E_{1/2,\ Red1}$ (V) vs. Fc/Fc$^+$	$E_{1/2,\ Red2}$ (V) vs. Fc/Fc$^+$
**1.H^{2+}**	−0.754 (72)	−1.158 (78)
**1.Se^{2+}**	−0.719 (74)	−1.196 (83)
**1.Te^{2+}**	−0.767 (73)	−1.207 (82)

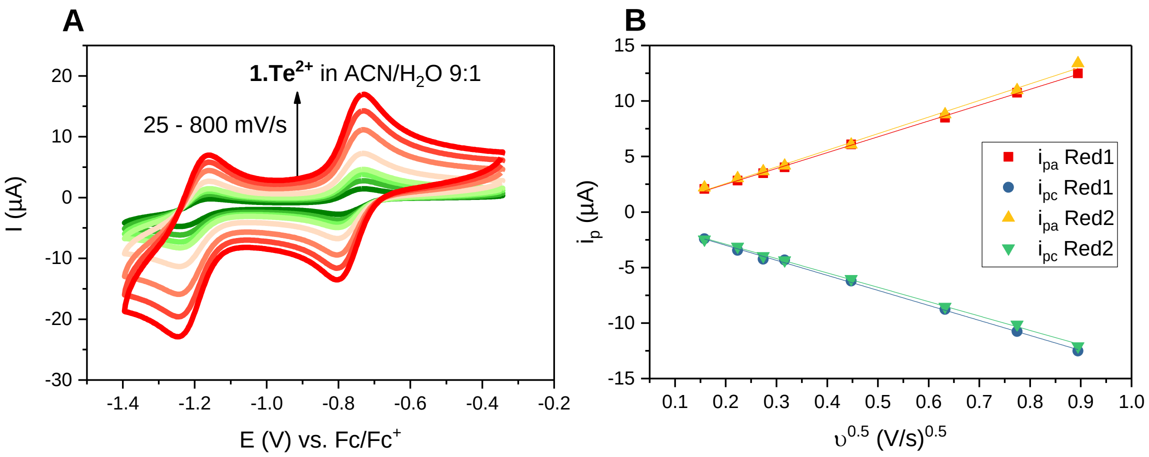

Figure S18. A) CVs of 0.25 mM **1.Te²⁺** in degassed ACN/H_2O 9:1, 100 mM $TBAPF_6$ at varying scan rates. B) The corresponding peak currents of both reductive couples as a function of the square-root of the scan rate ν including linear fits.

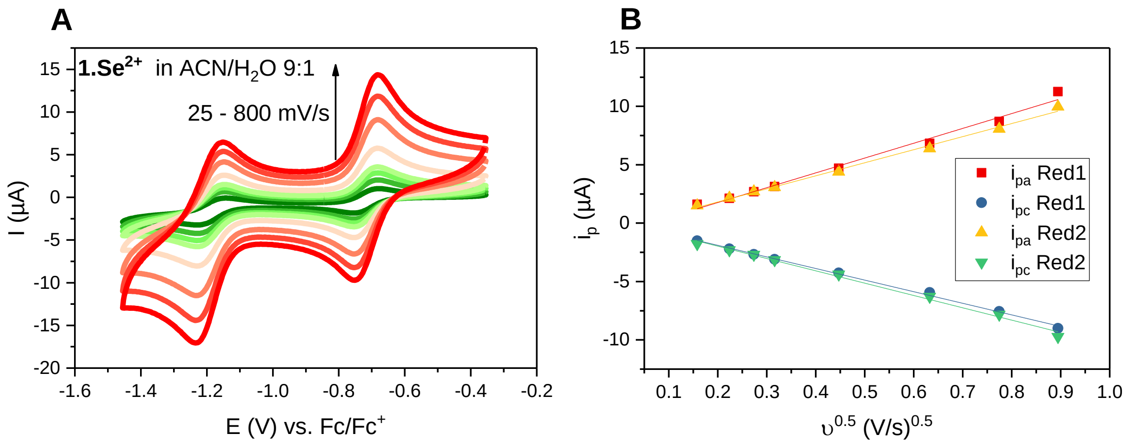

Figure S19. A) CVs of 0.25 mM **1.Se²⁺** in degassed ACN/H_2O 9:1, 100 mM $TBAPF_6$ at varying scan rates. B) The corresponding peak currents of both reductive couples as a function of the square-root of the scan rate ν including linear fits.

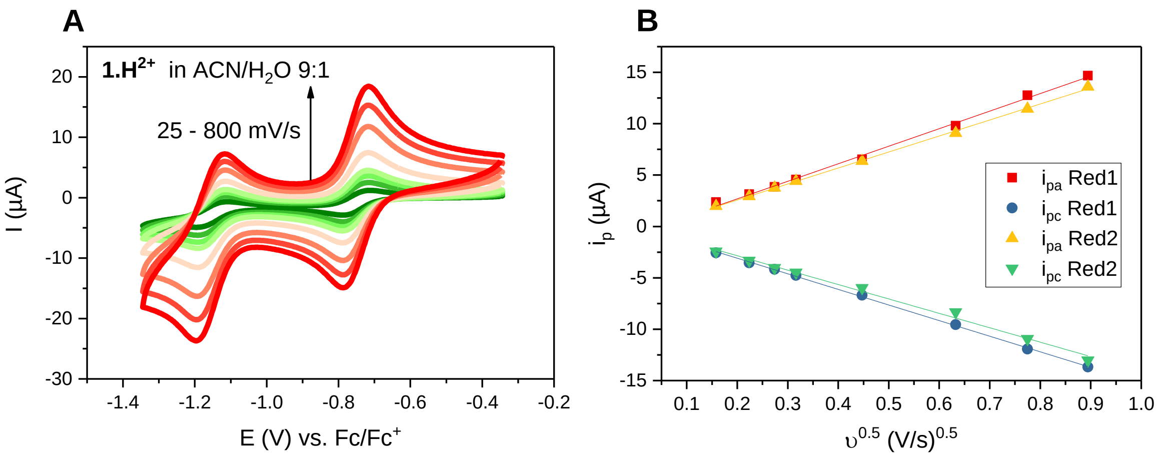

Figure S20. A) CVs of 0.25 mM **$1.H^{2+}$** in degassed ACN/H_2O 9:1, 100 mM $TBAPF_6$ at varying scan rates. B) The corresponding peak currents of both reductive couples as a function of the square-root of the scan rate ν including linear fits.

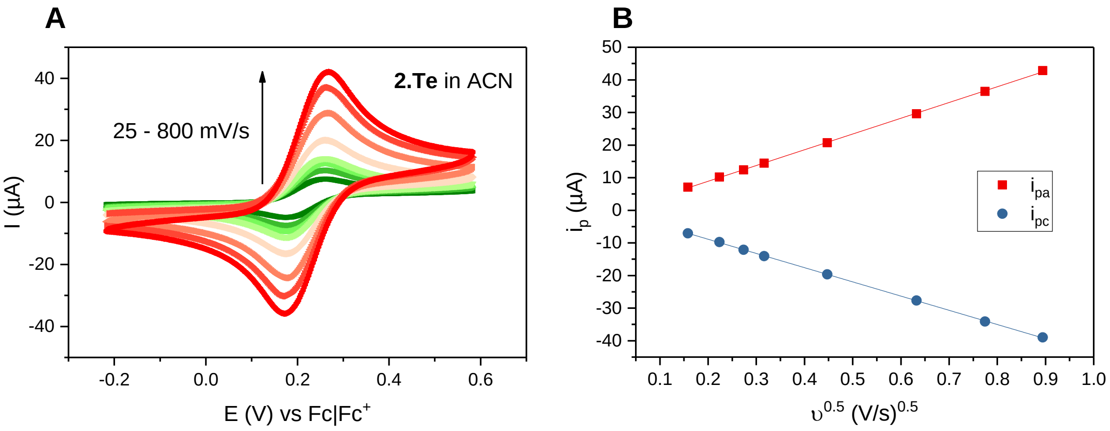

Figure S21. A) CVs at varying scan rate of 0.5 mM **2.Te** in ACN, 100 mM $TBAPF_6$. B) The associated anodic and cathodic peak currents as a function of the square-root of the scan rate including linear fits.

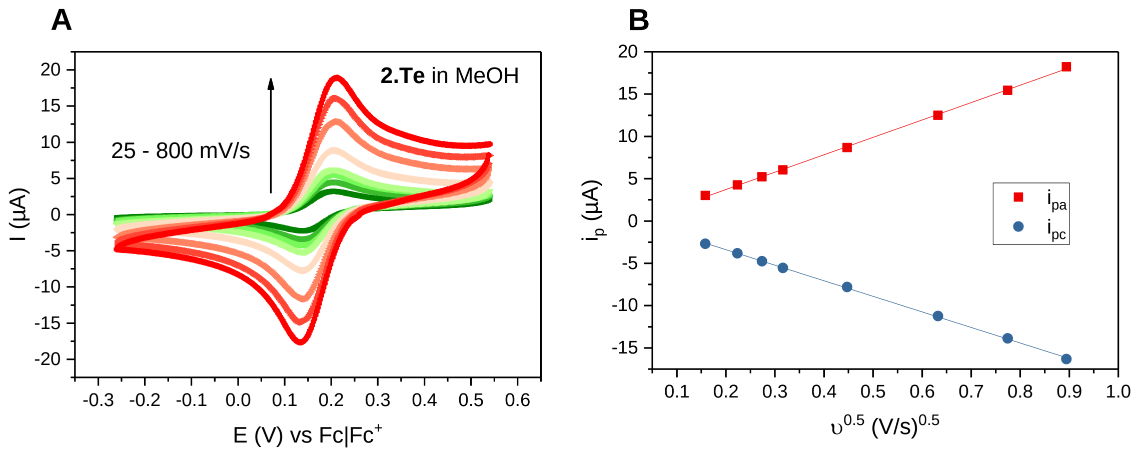

Figure S22. A) CVs at varying scan rate of 0.25 mM **2.Te** in MeOH, 100 mM $TBAClO_4$. B) The associated anodic and cathodic peak currents as a function of the square-root of the scan rate including linear fits.

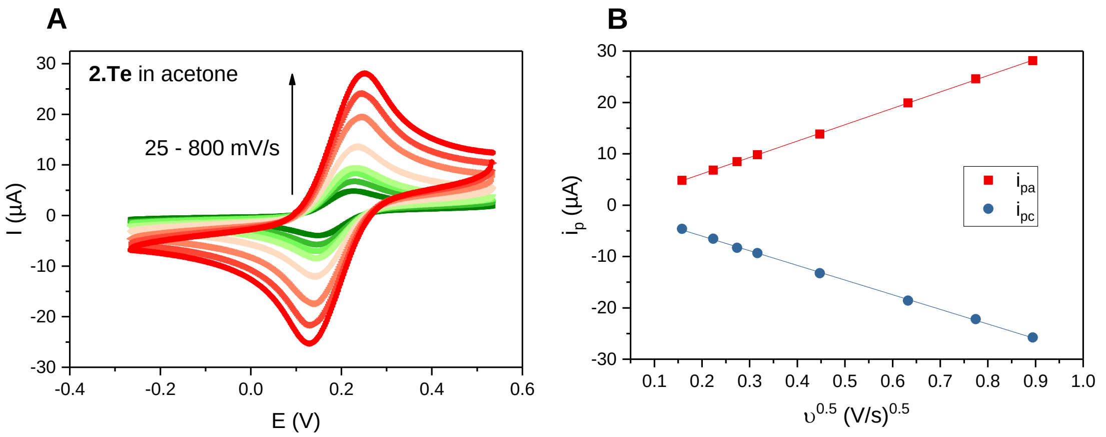

Figure S23. A) CVs at varying scan rate of 0.25 mM **2.Te** in acetone, 100 mM $TBAPF_6$. B) The associated anodic and cathodic peak currents as a function of the square-root of the scan rate including linear fits.

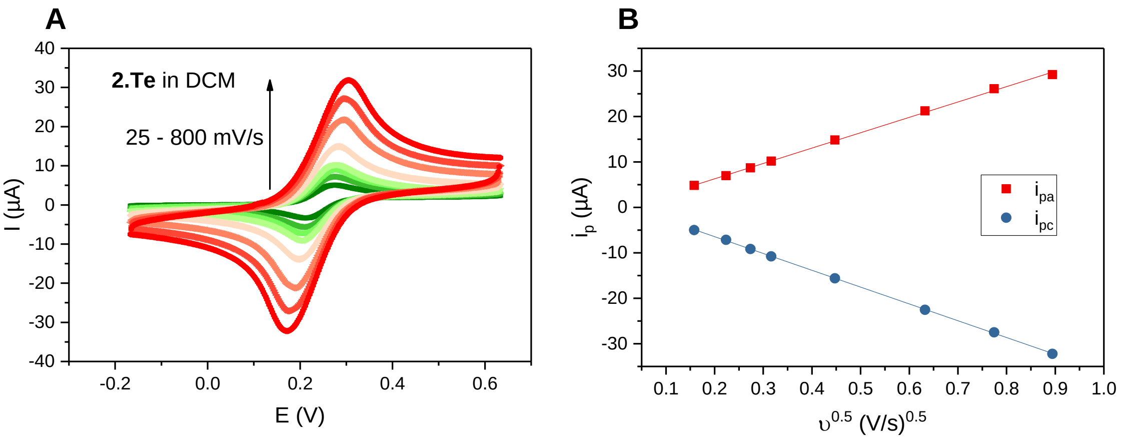

Figure S24. A) CVs at varying scan rate of 0.25 mM **2.Te** in DCM, 100 mM $TBAPF_6$. B) The associated anodic and cathodic peak currents as a function of the square-root of the scan rate including linear fits.

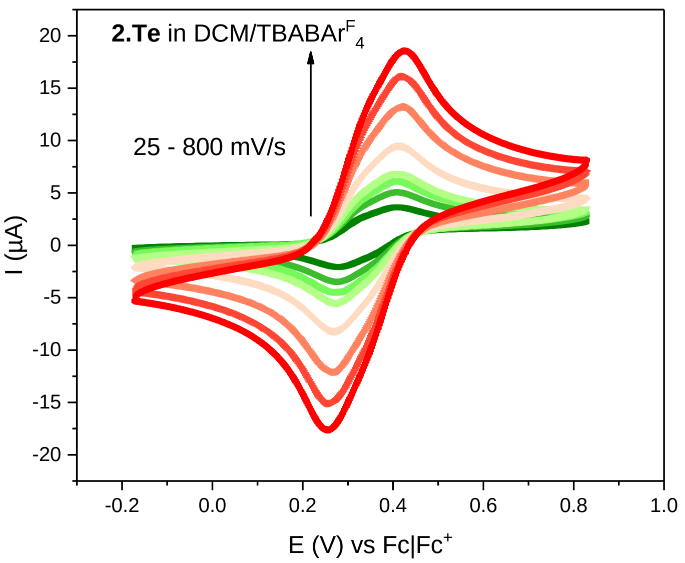

Figure S25. CVs at varying scan rate of 0.25 mM **2.Te** in DCM, 100 mM TBABAr$^F{}_4$.

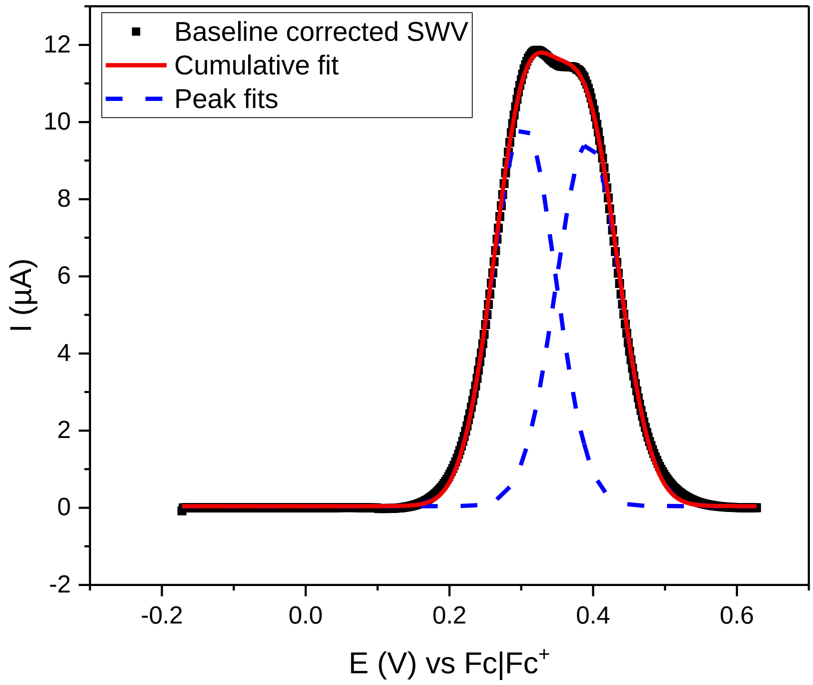

Figure S26. Baseline corrected SWV of 0.25 mM **2.Te** in DCM, 100 mM $TBABAr^F_4$ including Gaussian peak deconvolution.

Table S3. Half-wave potentials $E_{1/2}$ of **2.Te** in different solvents as assessed by SWV. In all cases 100 mM supporting electrolyte were used ($TBAPF_6$, unless otherwise indicated).

$E_{1/2}$ (V)	MeOH	Acetone	ACN	DCM	DCM[a]
**vs Fc|Fc+**	0.192	0.188	0.218	0.241	0.304, 0.393
**vs Ag|AgNO$_3$**	0.344	0.350	0.328	0.503	0.576, 0.665

a – with 100 mM $TBABAr^F_4$ electrolyte. Two Fc couples can be resolved, whose $E_{1/2}$ were obtained by deconvolution of the SWV (Figure S26).

5. Voltammetric Anion Binding Studies

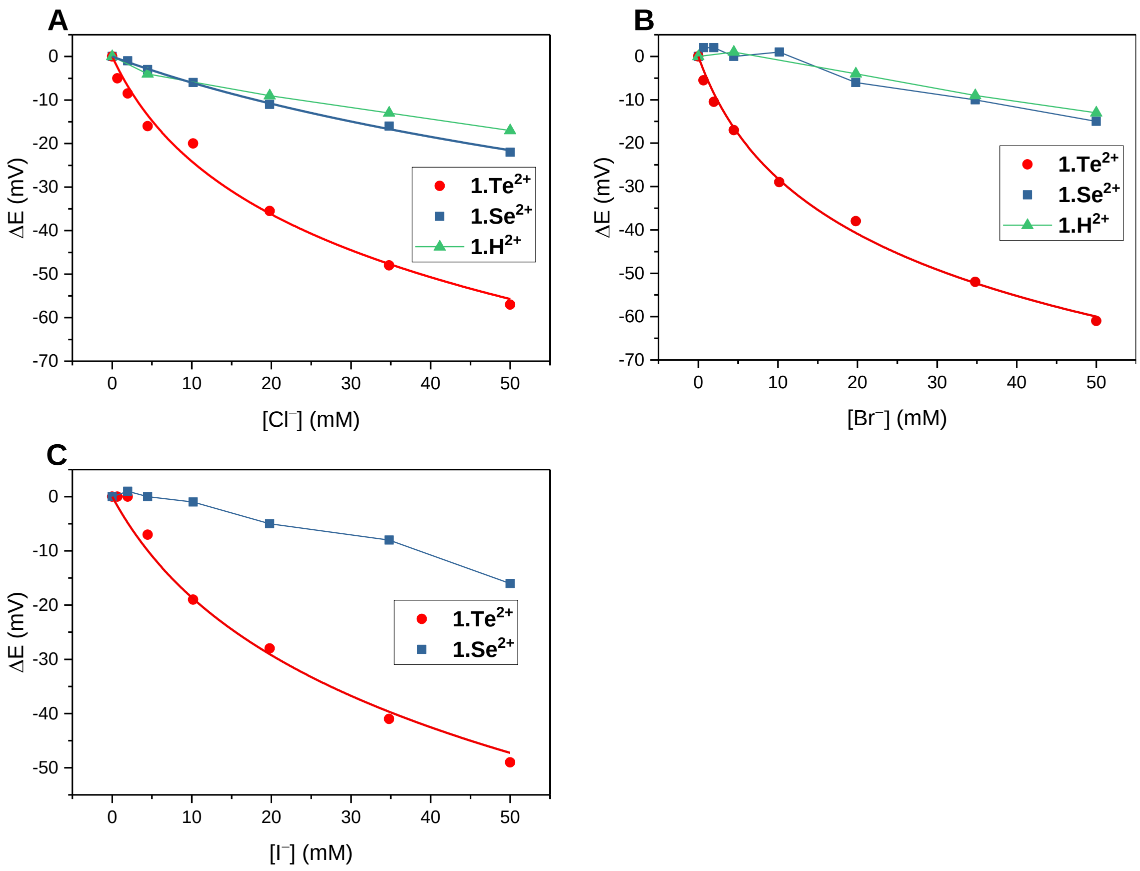

Figure S27. Cathodic voltammetric shifts of **1.Te^{2+}/Se^{2+}/H^{2+}** in ACN/H_2O 9:1 upon titration with A) Cl^-, B) Br^- and C) I^-. [receptor] = 0.1 mM with 100 mM $TBAPF_6$ supporting electrolyte. The overall ionic strength was kept constant at 100 mM throughout. Thick solid lines represent fits to a 1:1 host-guest Nernst model (eqn. 1) for **1.Te^{2+}** in all cases and for **1.Se^{2+}** upon titration with Cl^- in all other cases they are connecting lines to guide the eye only. Note the different y-axis scaling for the graphs.

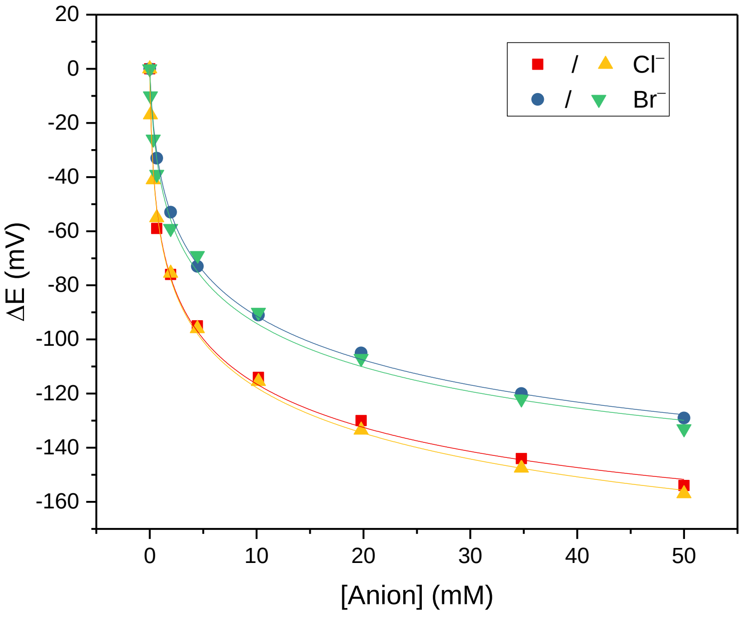

Figure S28. Cathodic voltammetric shifts of **2.Te** in ACN upon repeat titration with Cl^- or Br^-. [2.Te] = 0.1 mM with 100 mM $TBAPF_6$ supporting electrolyte. The overall ionic strength was kept constant at 100 mM throughout. Solid lines represent fits to a 1:1 host-guest Nernst model (eqn. 1).

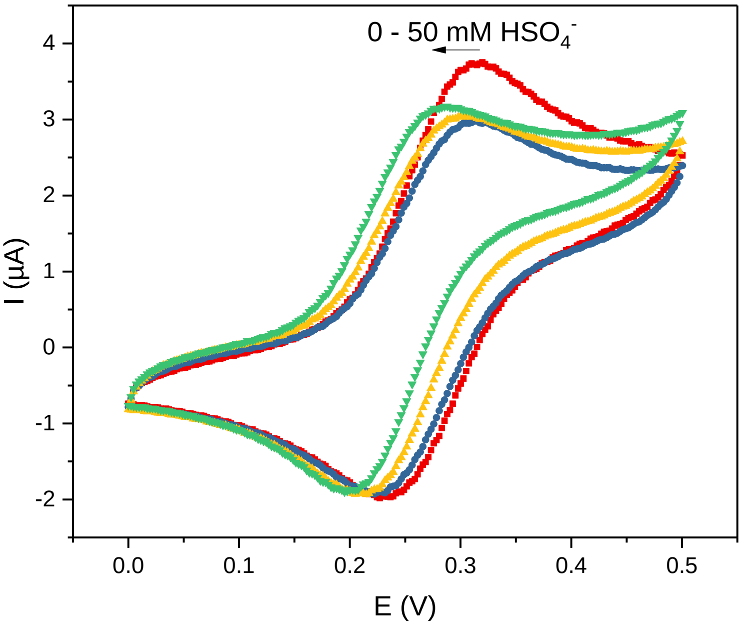

Figure S29. Representative evolution of CVs of 0.1 mM **2.Te** in ACN/H_2O 19:1, 100 mM $TBAPF_6$, upon addition of up to 50 mM HSO_4^-.

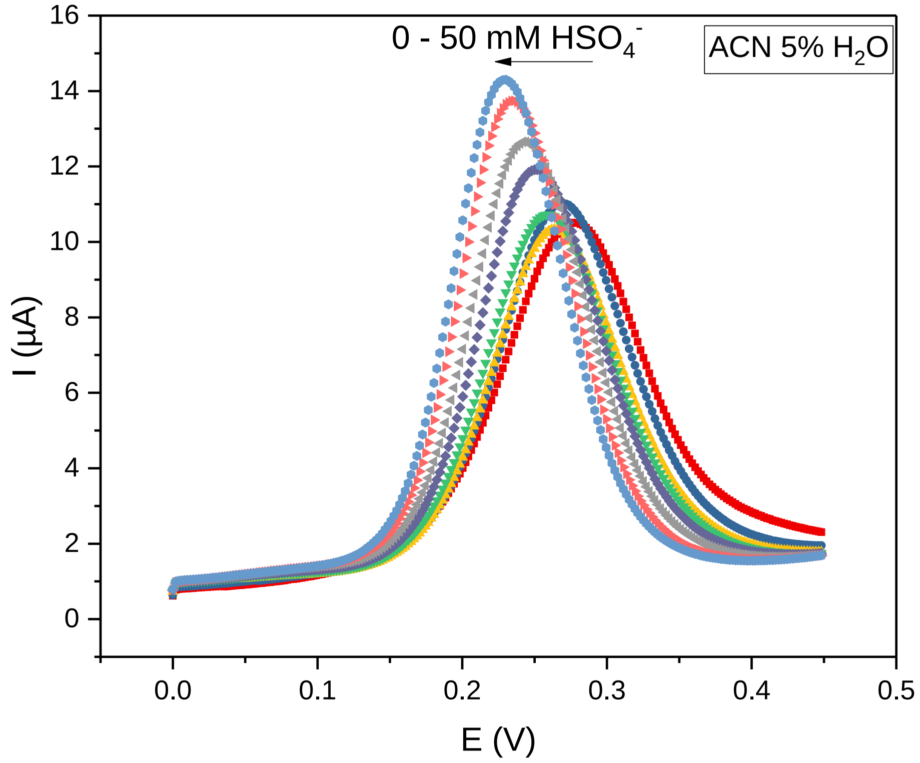

Figure S30. Representative evolution of SWVs of 0.1 mM **2.Te** in ACN/H_2O 19:1, 100 mM $TBAPF_6$, upon addition of up to 50 mM HSO_4^-.

6. Determination of Binding Enhancement Factors (BEFs)

For both **1.Te^{2+}/Se^{2+}/H^{2+}** and **2.Te** fitting of the voltammetric isotherms according to eqn. 1 afforded K_{Red} (corresponding to $K_{\mathbf{1.Te^{\bullet+}/Se^{\bullet+}}}$ or $K_{2.Te}$) values that were close or equal to 0 in all cases. As such, the determination of the magnitude of redox switch-OFF or -ON upon reduction/oxidation, i.e. the BEF = K_{Ox}/K_{Red} affords meaningless values. Instead all BEF values stated in the main text (e.g. Tables 2-3) were obtained via the more general eqn. S2 and ΔE_{max} (at $[A^-]$ = 50 mM).[6-7] Importantly, BEF determination via eqn. S2 is not inconsistent with a $K_{Red} \approx 0$. The interested reader is referred to references[6-7] for further details and related discussions.

$$\Delta \mathrm{E} = -\frac{RT}{nF}\ln\left(\frac{1+K_{Ox}[A^-]}{1+K_{Red}[A^-]}\right) \quad \text{eqn. 1}$$

$$\Delta \mathrm{E}_{max} = -\frac{RT}{nF}\ln\left(\frac{K_{Ox}}{K_{Red}}\right) = -0.059\,V * \log\left(\frac{K_{Ox}}{K_{Red}}\right) \quad \text{eqn. S2}$$

Which can be rearranged to:

$$BEF = \frac{K_{Ox}}{K_{Red}} = 10^{\frac{\Delta E_{max}}{-0.059\,V}} \quad \text{eqn. S2}$$

7. Receptor Comparisons

The chemical structure of the structurally related halogen bonding (XB) and hydrogen bonding (HB) receptors **5.XB/HB$_{SAM}$**, whose voltammetric anion sensing performance were published recently, is shown in Figure S31.[8]

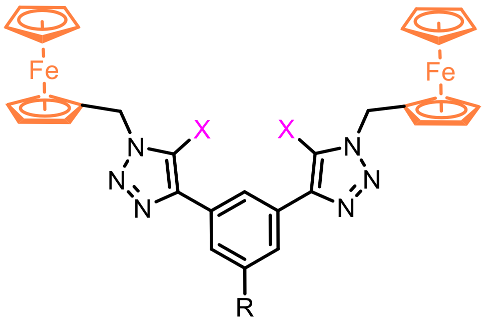

5.XB/HB: X = I/H

Figure S31. Chemical structures of **5.XB/HB**.

As shown in Figures S32-S33, **2.Te** outperforms both **5.XB** and **5.HB** as a voltammetric sensor towards most anions, in particular towards the halides in ACN and ACN/H_2O 19:1. Similarly, **1.Te^{2+}** is a very competitive halide sensor in ACN/H_2O 9:1 (Figure S34).

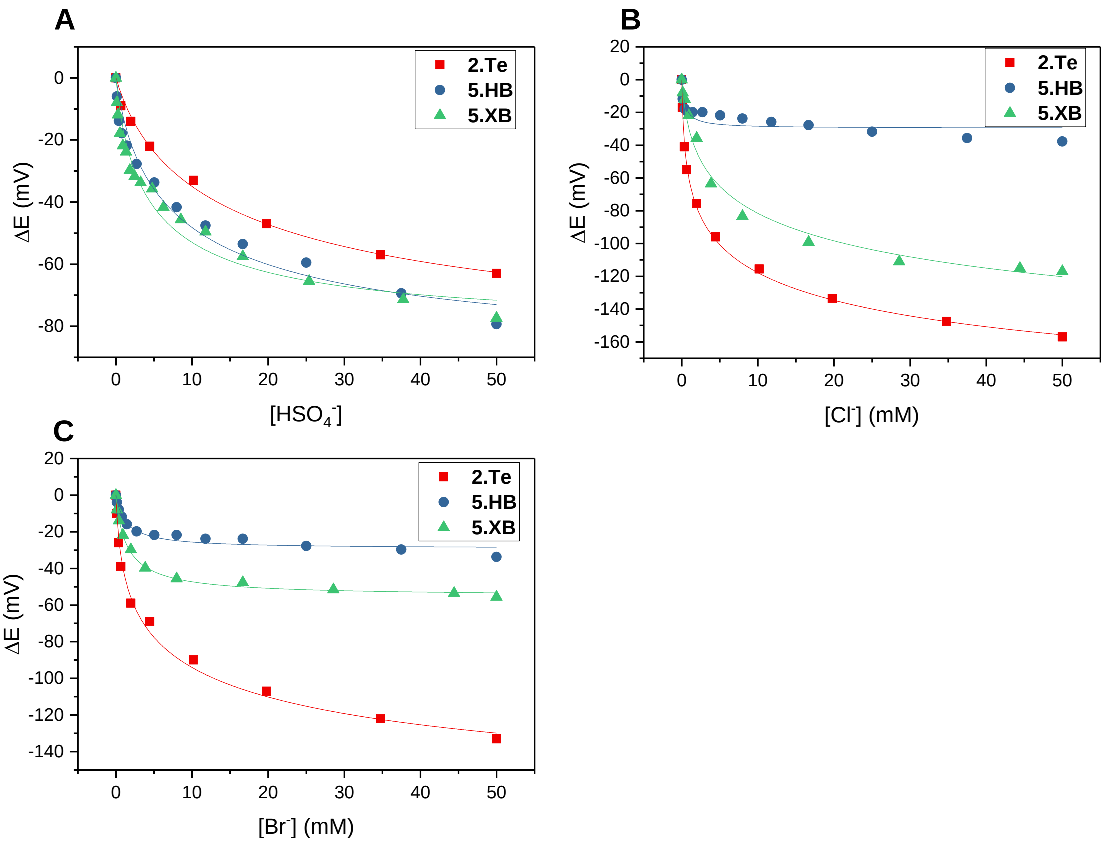

Figure S32. Cathodic voltammetric shifts of **2.Te** and **5.XB/HB** in ACN upon titration with A) HSO_4^-, B) Cl^- and C) Br^-. [receptor] = 0.1 mM with 100 mM $TBAPF_6$ supporting electrolyte for **2.Te** and 100 mM $TBAClO_4$ for **5.XB/HB**. The overall ionic strength was kept constant at 100 mM throughout. Solid lines represent fits to a 1:1 host-guest Nernst model (eqn. 1). Note the different y-axis scaling for the graphs.

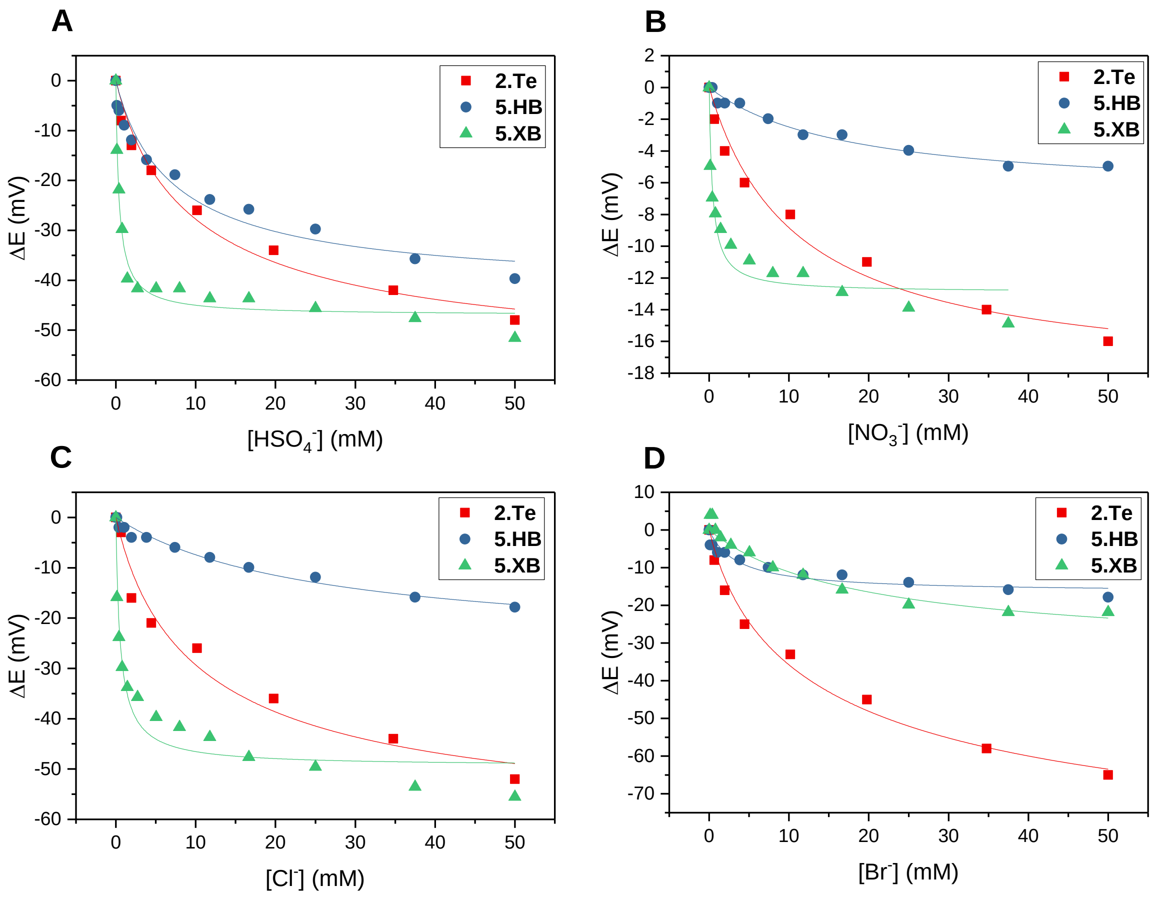

Figure S33. Cathodic voltammetric shifts of **2.Te** and **5.XB/HB** in ACN/H_2O 19:1 upon titration with A) HSO_4^-, B) NO_3^-, C) Cl^- and D) Br^-. [receptor] = 0.1 mM with 100 mM $TBAPF_6$ supporting electrolyte for **2.Te** and 100 mM $TBAClO_4$ for **5.XB/HB**. The overall ionic strength was kept constant at 100 mM throughout. Solid lines represent fits to a 1:1 host-guest Nernst model (eqn. 1). Note the different y-axis scaling for the graphs.

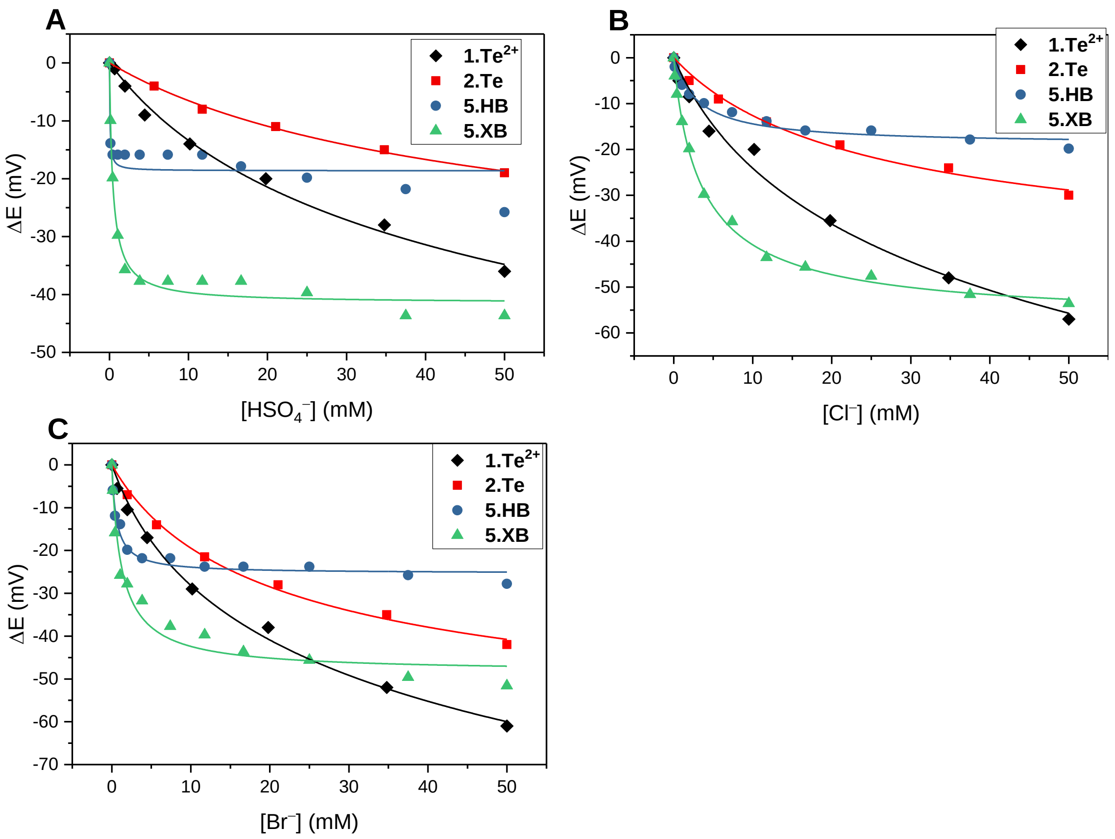

Figure S34. Cathodic voltammetric shifts of **1.Te^{2+}**, **2.Te** and **5.XB/HB** in ACN/H_2O 9:1 upon titration with A) HSO_4^-, B) Cl^- and C) Br^-. [receptor] = 0.1 mM with 100 mM $TBAPF_6$ supporting electrolyte for 1.Te^{2+} and **2.Te** and 100 mM $TBAClO_4$ for **5.XB/HB**. The overall ionic strength was kept constant at 100 mM throughout. Solid lines represent fits to a 1:1 host-guest Nernst model (eqn. 1). Note the different y-axis scaling for the graphs.

8. References

1. Thordarson, P., Determining association constants from titration experiments in supramolecular chemistry. *Chem. Soc. Rev.* **2011,** *40* (3), 1305-1323.
2. Li, G.; Xu, L.; Zhang, W.; Zhou, K.; Ding, Y.; Liu, F.; He, X.; He, G., Narrow-Bandgap Chalcogenoviologens for Electrochromism and Visible-Light-Driven Hydrogen Evolution. *Angew. Chem. Int. Ed.* **2018,** *57* (18), 4897-4901.
3. Jin, L.-M.; Xu, X.; Lu, H.; Cui, X.; Wojtas, L.; Zhang, X. P., Effective Synthesis of Chiral N-Fluoroaryl Aziridines through Enantioselective Aziridination of Alkenes with Fluoroaryl Azides. *Angew. Chem. Int. Ed.* **2013,** *52* (20), 5309-5313.
4. Mathur, P.; Tauqeer, M.; Torubaev, Y. V.; Shaikh, M. M.; Lahiri, G. K.; Pasynskii, A. A.; Pavlova, A. V., Step-by-step transformations of ferrocenyltellurium complexes of Group VIB metal carbonyls. *J. Organomet. Chem.* **2014,** *758*, 55-59.
5. Docker, A.; Guthrie, C. H.; Kuhn, H.; Beer, P. D., Modulating Chalcogen Bonding and Halogen Bonding Sigma-Hole Donor Atom Potency and Selectivity for Halide Anion Recognition. *Angew. Chem. Int. Ed. Engl.* **2021,** *60* (40), 21973-21978.
6. Hein, R.; Beer, P. D.; Davis, J. J., Electrochemical Anion Sensing: Supramolecular Approaches. *Chem. Rev.* **2020,** *120* (3), 1888-1935.
7. Hein, R.; Li, X.; Beer, P. D.; Davis, J. J., Enhanced voltammetric anion sensing at halogen and hydrogen bonding ferrocenyl SAMs. *Chem. Sci.* **2021,** *12* (7), 2433-2440.

8. Patrick, S. C.; Hein, R.; Docker, A.; Beer, P. D.; Davis, J. J., Solvent Effects in Halogen and Hydrogen Bonding Mediated Electrochemical Anion Sensing in Aqueous Solution and at Interfaces. *Chem. Eur. J.* **2021,** *27* (39), 10201-10209.
